# Tracing TET1 expression in prostate cancer: discovery of malignant cells with a distinct oncogenic signature

**DOI:** 10.1186/s13148-021-01201-7

**Published:** 2021-11-29

**Authors:** U. Schagdarsurengin, C. Luo, H. Slanina, D. Sheridan, S. Füssel, N. Böğürcü-Seidel, S. Gattenloehner, G. B. Baretton, L. C. Hofbauer, F. Wagenlehner, T. Dansranjav

**Affiliations:** 1 Clinic of Urology, Pediatric Urology and Andrology, Justus-Liebig-University Giessen, Giessen, Germany; 2 Working Group Epigenetics of Urogenital System, Clinic of Urology, Pediatric Urology and Andrology, Justus-Liebig-University Giessen, Giessen, Germany; 3 Institute of Medical Virology, Justus-Liebig-University Giessen, Giessen, Germany; 4 Institute of Pathology, Justus-Liebig-University Giessen, Giessen, Germany; 5 Department of Urology, University Hospital and Faculty of Medicine, Technische Universität Dresden, Dresden, Germany; 6 Institute of Neuropathology, Justus-Liebig-University Giessen, Giessen, Germany; 7 Institute of Pathology, University Hospital and Faculty of Medicine, Technische Universität Dresden, Dresden, Germany; 8 Division of Endocrinology, Diabetes, and Bone Diseases, Department of Medicine III and University Center for Healthy Aging, Technische Universität Dresden, Dresden, Germany

**Keywords:** Prostate cancer, TET1, Oncogene, ZNF transcription factor, ZNF antiviral protein

## Abstract

**Background:**

Ten–eleven translocation methylcytosine dioxygenase 1 (TET1) is involved in DNA demethylation and transcriptional regulation, plays a key role in the maintenance of stem cell pluripotency, and is dysregulated in malignant cells. The identification of cancer stem cells (CSCs) driving tumor growth and metastasis is the primary objective of biomarker discovery in aggressive prostate cancer (PCa). In this context, we analyzed TET1 expression in PCa.

**Methods:**

A large-scale immunohistochemical analysis of TET1 was performed in normal prostate (NOR) and PCa using conventional slides (50 PCa specimens) and tissue microarrays (669 NOR and 1371 PCa tissue cores from 371 PCa specimens). Western blotting, RT-qPCR, and 450 K methylation array analyses were performed on PCa cell lines. Genome-wide correlation, gene regulatory network, and functional genomics studies were performed using publicly available data sources and bioinformatics tools.

**Results:**

In NOR, TET1 was exclusively expressed in normal cytokeratin 903 (CK903)–positive basal cells. In PCa, TET1 was frequently detected in alpha-methylacyl-CoA racemase (AMACR)–positive tumor cell clusters and was detectable at all tumor stages and Gleason scores. Pearson’s correlation analyses of PCa revealed 626 *TET1*-coactivated genes (*r* > 0.5) primarily encoding chromatin remodeling and mitotic factors. Moreover, signaling pathways regulating antiviral processes (62 zinc finger, ZNF, antiviral proteins) and the pluripotency of stem cells were activated. A significant proportion of detected genes exhibited *TET1*-correlated promoter hypomethylation. There were 161 genes encoding transcription factors (TFs), of which 133 were ZNF-TFs with promoter binding sites in *TET1* and in the vast majority of *TET1*-coactivated genes.

**Conclusions:**

TET1-expressing cells are an integral part of PCa and may represent CSCs with oncogenic potential.

**Supplementary Information:**

The online version contains supplementary material available at 10.1186/s13148-021-01201-7.

## Background

Ten–eleven translocation (TET) family dioxygenases are the main catalysts responsible for the oxidation of 5-methylcytosine (5mC) in DNA to 5-hydroxymethylcytosine (5hmC) and further oxidation products. TET DNA demethylases and their catalytic products are key regulators of embryonic development, stem cell functions, and lineage specification [1]. Safeguarding bivalent promoters from de novo methylation is one of the key functions of TETs in stem cells, and a number of chromatin immunoprecipitation (ChIP) and knock-out experiments in human and mouse embryonic stem cells have demonstrated that in particular, TET1 and Tet1, respectively, bind to hypomethylated promoters of pluripotency-related genes and maintain their active status [2–5]. The essential role of TET1/Tet1 in maintaining pluripotency and regulating differentiation has been shown in different stem cell types, for example, embryonic [5, 6], neural [7], mesenchymal [8], trophoblast [9, 10], and hematopoietic stem cells [11].

In normal prostate epithelium, the stem cell population is located in the proliferative basal cell compartment. The self-renewing basal stem cells give rise to bipotent basal progenitors which are precursor cells of prostatic secretory (luminal) cells [12]. In PCa, cancer stem cells (CSCs) capable of self-renewal and asymmetric division and having adaptive capacity have been postulated to be responsible for tumor growth and metastasis [13–15], tumor survival after chemotherapeutics and radiation, and initiation of therapeutic relapse [16–18]. Despite basic common features attributed to cell stemness, CSCs in PCa exhibit a pronounced molecular heterogeneity displayed in a large number of extracellular and intracellular markers [15]. Thus, in order to develop therapeutic strategies for PCa, there is still great interest in deeper understanding of molecular mechanisms responsible for establishment and maintenance of CSCs.

The balance between 5mC and unmethylated cytosine is needed in order to maintain stem cell self-renewal as well as upon cell differentiation [2, 19, 20]. In human malignancies, TET1 has been identified as a fusion partner of the mixed-lineage leukemia gene in acute myeloid leukemia [21]. Dysregulation of TET1 expression or function and aberrant methylation have been observed in a wide range of cancers, for example, gastric cancer, thyroid carcinoma, lung cancer, gliomas, esophageal carcinoma, and breast cancer [22–27]. With regard to PCa, data are still scarce. In PCa tissues, TET1 has been found to be downregulated, and in xenograft models, TET1 depletion facilitates tumor growth and PCa metastasis [28]. Furthermore, in high-risk PCa, *TET1* mutation and mRNA downregulation are associated with worse metastasis-free survival [29]. However, existing studies on TET1 comprise a relatively low number of primary PCa specimens, and the role of TET1-expressing cells in PCa remains unclear.

Here, we describe a large-scale TET1 protein expression study in normal prostate (NOR) and PCa, and a genome-wide correlation and regulatory network study using different publicly accessible data sources and bioinformatics tools. Our data demonstrate striking differences in the TET1 expression profiles of NOR and PCa, clarify the epigenetic background of TET1-upregulation in PCa, identify all *TET1*-coactivated genes and demethylated promoters, and provide their functional characterization. All in all, our data suggest that TET1-expressing cell clusters in PCa represent CSCs with distinct oncogenic potential.

## Methods

### Immunohistochemical analysis of TET1 expression

Conventional slides from formalin-fixed, paraffin-embedded tissue blocks were prepared from specimens obtained from 50 PCa patients who underwent radical prostatectomy at the Department of Urology, Pediatric Urology and Andrology (Justus-Liebig-University Giessen, Germany). All patients gave their written consent, and the study was approved by the ethics committee of the Medical Faculty (Justus-Liebig-University Giessen, Germany) (Ethics Vote: 49/05). All tissue samples were examined and characterized at the Institute of Pathology (Justus-Liebig-University Giessen, Germany). The PCa specimens were characterized with regard to the pathologic stage, differentiation grade (Gleason score), presence of lymph node metastases, and residual tumor (Table 1). Tissue microarrays (TMA) originating from formalin-fixed, paraffin-embedded tissue blocks of 371 radical prostatectomy specimens were obtained from the Department of Urology of Technical University Dresden (Table 2). For each PCa patient, the TMA included four tissue cores from cancerous areas (PCa-TMA) and two tissue cores from non-cancerous areas (NOR-TMA).

**Table 1:** Clinical data of 50 PCa patients analyzed by IHC using conventional paraffin slides with regard to TET1 expression (*n* = 50 patients in total)

	Count (%)	Age (y)		PSA (ng/mL)	
		Median	Range	Median	Range
T2a–c	26 (52.0%)	64.24	47.8–75.1	7.0	0.1–54.8
T3a–b	24 (48.0%)	64.16	55.5–74.9	9.0	0.6–27.2
GS6	23 (46.0%)	65.88	47.8–75.1	8.8	0.1–24.8
GS7	20 (40.0%)	63.93	54.8–73.1	8.8	0.8–54.8
GS8	2 (4.0%)	65.53	58.2–72.8	6.0	2.7–9.3
GS9	5 (10.0%)	65.20	55.5–74.9	14.1	6–22.1

*PCa* prostate cancer, *ICH* immunohistochemical analysis, *TET1* ten–eleven translocation family member 1, *y* years, *PSA* prostate-specific antigen, *T* tumor stage, *GS* Gleason score

**Table 2:** Clinical data of PCa patients analyzed by IHC using TMA with regard to TET1 expression (*n* = 371 patients in total)

	Count (%)	Age (y)		PSA (ng/mL)	
		Median	Range	Median	Range
Low risk	97 (26.2%)	64.6	48.5–78.3	5.74	0.2–38.4
Intermed. risk	45 (12.1%)	64.9	50.6–76.9	12.9	2.9–83.8
High risk	229 (61.7%)	65.5	46.7–77.4	9.9	0.3–113
T2a–c	165 (44.5%)	65.1	46.7–78.3	7.2	0.2–45.1
T3a-b	144 (38.8%)	65.9	54.1–77.4	8.7	0.3–74.8
T4	62 (16.7%)	63.2	50.6–76.5	15.8	2.8–113
GS ≤ 6	73 (19.7%)	64.3	48.5–77.1	5.6	0.2–83.8
GS7	69 (18.6%)	65.6	50.6–78.3	8.0	2.9–50
GS8	147 (39.6%)	66.1	46.7–77.4	10.5	0.3–113
GS ≥ 9	82 (22.1%)	64.6	51.1–77.1	8.8	0.3–65.5

*PCa* prostate cancer, *IHC* immunohistochemical analysis, *TMA* tissue microarray, *TET1* ten–eleven translocation family member 1, *y* years, *PSA* prostate-specific antigen, *T* tumor stage, *GS* Gleason score, *Intermed. risk* intermediate risk

For immunohistochemical studies (IHC), slides of paraffin-embedded tissue were deparaffinized using xylene and then rehydrated by successive incubation in 100%, 96%, and 70% ethyl alcohol and then ddH_2_O. Next, slides were immersed in 1% hydrogen peroxide (H_2_O_2_) for 20 min to block endogenous peroxidase activity. Sections were washed once in 1X Tris–HCl buffer. For antigen retrieval, sections were incubated in citrate buffer (pH 6.0) and heated in a water bath at 70 °C for 15 min. Subsequently, sections were incubated overnight at 4 °C with the primary antibodies diluted in 0.1% non-fat milk in 1X Tris–HCl buffer. We characterized prostate cells on conventional slides using antibodies to TET1 (anti-TET1 rabbit polyclonal antibody, 1:400, GeneTex, GTX124207), the prostate basal cell marker cytokeratin 903 (CK903) (anti-34betaE12, 5 µg/mL, Leica Biosystems, Germany) and the prostate cancer cell marker alpha-methylacyl-CoA racemase (AMACR) (anti-AMACR, 10 µg/mL, Dako, Germany). After washing with 2% Triton/PBS buffer, the slides were incubated with biotinylated secondary antibodies (anti-rabbit IgG antibody, 1:500; anti-mouse IgG antibody, 1:1000; Dako, Germany) for 1 h at room temperature and washed three times with PBS buffer for 5 min. Next, slides were incubated for 30 min with an avidin–biotin complex system (Vector Laboratories, Burlingame, CA, USA), and bound antibody was detected using the 3-amino-9-ethylcarbazole substrate. Conventional slides were analyzed using an Olympus BX43 light microscope, and TMA slides were analyzed using ZEN 2.3 (blue edition) software after scanning with a Zeiss Axio Scan.Z1 slide scanner (Zeiss Microscopy).

### Cell culture

Human PCa cell lines PC3, LNCaP, and DU145 were obtained from the German Resource Center for Biological Material (DSMZ, Braunschweig, Germany). DU145 and LNCaP were cultured in RPMI 1640 medium (Gibco), and PC3 in DMEM (Gibco) supplemented with 10% fetal bovine serum (Gibco) and 1% penicillin/streptomycin (Gibco). Cells were cultured in 10-cm dishes in 5% CO_2_ at 37 °C to 90–100% confluency, then used for western blot, mRNA expression, and genome-wide methylation analyses.

### Analysis of TET1 expression in PCa cell lines by western blot

For protein extraction, PC3, DU145, and LNCaP cells were washed with PBS buffer and lysed in lysis buffer (10 mM Tris–HCl, 150 mM NaCl, 1 mM EDTA, 1 mM DTT, 1 mM phenylmethylsulfonylfluoride, and 1% Triton X-100, pH 7.4) supplemented with complete protease inhibitor cocktail tablets (Roche). After an incubation on ice for 30 min, cell debris was removed by centrifugation at 12,000 g for 20 min, and protein was quantified using the Pierce BCA protein assay (Thermo Fisher). In total, 10 μg of protein was mixed with 5 µL loading buffer (4.5 µL Laemmli buffer and 0.5 µL *ß*-mercaptoethanol), heated at 95 °C for 5 min, and separated in a polyacrylamide gel (resolving gel 7.5%, stacking gel 4%). Semi-dry transfer (Trans-Blot SD Semi-dry transfer cell, Bio-Rad) was performed for 1 h at 150 mA (max. 25 V) onto polyvinylidene-difluoride membranes (Merck-Millipore). Blocking was performed using Odyssey blocking buffer (1:3 diluted in PBS) for 1 h at room temperature. The membranes were incubated overnight at 4 °C with TET1 (1:2000, GeneTex, GTX124207) and GAPDH antibodies (1:2500, Abcam, ab9485) diluted in Odyssey blocking buffer (1:3 diluted in PBS, plus 0.1% Tween-20). Membranes were washed in PBS-Tween-20 buffer (0.1% Tween-20 in PBS) and incubated with IRDye-conjugated secondary antibodies (LI-COR Biosciences) for 1 h at room temperature. After washing in PBS-Tween-20 buffer, the membranes were rinsed with PBS and the fluorescence signals were detected using the Odyssey Fc imaging system.

### Analysis of *TET1* expression in PCa cell lines by RT-qPCR

The total RNA was extracted from PC3, DU145, and LNCaP cells using the peqGOLD TriFast reagent (VWR) according to manufacturer’s instructions. Reverse transcription was performed on 1 µg total RNA using M-MLV transcriptase and adjusted buffer system (Promega), random hexamers, and poly-dT primers for 1 h at 42 °C. The resulting cDNAs were purified using a QIAquick PCR purification kit (Qiagen), and the concentrations were measured. Real-time PCRs were performed using 50 ng of cDNA per PCR in a Rotor-Gene Q PCR Cycler (Qiagen) for *TET1* (forward primer: 5′-TCCTGGTGCTATTCCAGTCC-3′, reverse primer: 5′-CAGGAAGGAAGACAGGCAAG-3′, product size: 110 base pairs) with the reference gene *GAPDH* (forward primer: 5′-TGGAGAAGGCTGGGGCTCAT-3′, reverse primer: 5′-GACCTTGGCCAGGGGTGCTA-3′, product size: 176 base pairs). *TET1* expression was calculated as a relative expression by normalization to *GAPDH* using the 2^−∆∆Ct^ method.

### Genome-wide methylation analyses in PCa cell lines

Genome-wide methylation analysis of PC3, DU145, and LNCaP cells was performed using the Illumina Infinium HumanMethylation 450 K BeadChip platform provided by Life & Brain GmbH. The DNA was extracted and purified from PCa cell lines using a DNA Mini kit (Qiagen), and 1 µg of DNA was used for each methylation analysis. Import of raw Illumina 450 K BeadChip IDAT files, data preprocessing, and correction and normalization steps were carried out using the R computing environment (v. 4.0) with the Bioconductor package “minfi” (v. 1.36.0). The methylation score of each CpG was represented as a beta(*β*)-value.

### Analysis of viral infections in PCa and BPH tissues

Tissue samples from PCa and BPH were tested for the human cytomegalovirus (HCMV), Epstein–Barr virus (EBV), herpes simplex viruses 1 and 2 (HSV1/2), and JC and BK viruses. The DNA of HCMV and EBV was positively tested and further analyzed in 34 PCa and 16 BPH specimens. Approximately, 10 to 25 µg DNA was used as a template for the artus CMV-LC-PCR and artus EBV-LC-PCR kit (Qiagen), respectively. PCRs were performed according to the manufacturer's instructions and calibrated against the WHO first International Standard for Human Cytomegalovirus (NIBSC, code 09/162), and the WHO first International Standard for Epstein–Barr Virus (NIBSC, code 09/260), detecting a quantifiable minimum of 80 genomes/mL for HCMV and 100 genomes/mL for EBV. Positive PCR results below these thresholds were therefore only reported as < 80 or < 100 positive. An internal control excluded inhibitory compounds in the sample.

### Data sources, application, and bioinformatic evaluation

To explore the molecular background of PCa specimens exhibiting high TET1 expression, we employed the Cancer Genome Atlas (TCGA) database (tcgaportal.org), in particular the Illumina Infinium HumanMethylation 450 K BeadChip data and genome-wide RNA-sequencing data of 341 PCa (adenocarcinoma) and 35 NOR. Supplemental clinical data, raw methylation data (“IDAT” files), and RNA-seq data (“HTSeq-counts” files) were extracted from TCGA using the R package “TCGAbiolinks” (v. 2.18.0). Data preprocessing and subsequent analyses were performed using the R packages “minfi” (v. 1.36.0) for Illumina 450 K data and “Biobase” (v.2.50) for RNA sequence data. Differential expression and differential methylation analyses were performed using the eBayes function in the “limma” package. *p* values were adjusted for multiple comparisons using the Benjamini–Hochberg method.

Methylation (*ß*) values of all *TET1* CpG probes in TCGA (in total 30) covering the promoter, 5′-untranslated region (5′-UTR), and gene body of *TET1* were correlated to *TET1* expression using data from 341 PCa and 35 NOR, using Spearman’s correlation. All differentially methylated *TET1* CpG probes in PCa versus NOR were evaluated (Mann–Whitney U test). The PCa specimens from TCGA were divided according to *TET1* expression and promoter methylation levels into three groups: “*TET1*-high” (expression > 85th percentile, hypomethylated, *n* = 51), “*TET1*-low” (expression < 40th percentile, hypermethylated, *n* = 136) and “*TET1*-moderate” (intermediate expression and methylation). All differentially methylated *TET1* CpG probes in *TET1*-high versus *TET1*-low PCa were evaluated (Mann–Whitney U test). Using 341 PCa, *TET1* expression was tested for correlation with the expression of all protein-encoding genes (Pearson’s correlation), and genes showing strong positive correlation (referred as *TET1*-coactivated genes) were further analyzed with regard to promoter methylation in *TET1*-high versus *TET1*-low PCa (Mann–Whitney U test). The statistical analyses are described in detail below.

The Human Transcription Factors databank (http://humantfs.ccbr.utoronto.ca/index.php) [30] was used to identify *TET1*-coactivated genes encoding transcription factors (TFs). JASPAR 2020, a database of curated non-redundant TF-binding profiles stored as position frequency matrices (jaspar2020.genereg.net), was used to analyze the binding scores and binding motifs of TFs. Promoter sequences (the 1500 base pairs upstream from the transcription start site, TSS1500) of *TET1* and *TET1*-coactivated genes were extracted and scanned for binding sites for *TET1*-coactivated TFs. The significance (*p* values) of TF-binding sites (TF-BSs) was calculated using the R package “TFMPvalue” (v. 0.0.8) based on the method described by Touzet and Varre [31], and statistically significant TF-BSs (*p* < 0.01) were considered true. Transcription regulatory modules (TRMs) were identified using the R package “rTRM” (v. 1.28.0) [32].

Functional genomics studies were performed on *TET1*-coactivated genes in PCa. Gene ontology (GO) analysis was done using the Database for Annotation, Visualization and Integrated Discovery (DAVID) [33, 34]. Gene set enrichment analysis (GSEA) was done using the Molecular Signatures Database (MSigDB) [35]. Pathway analysis was done using the Kyoto Encyclopedia of Genes and Genomes (KEGG) [36]. Protein–protein interaction (PPI) and PPI network (interactome) analyses were done on *TET1*-coactivated TFs using the BioGRID database of protein, genetic, and chemical interactions [37].

In order to assess whether *TET1*-coactivated genes in PCa may also possess TET1-binding sites in their promoters, we selected Tet1 ChIP-seq data generated on mouse trophoblast stem cells (GSE109545) [9]. Genes having Tet1-binding sites in their TSS1500 promoters were considered for further analysis.

### Statistical analyses

The R function “rcorr” from the “Hmisc” package was used for correlation analyses. Correlation coefficients between *TET1* expression and the expression of 19,252 protein-coding genes were calculated for 341 PCa using Pearson’s method, and genes showing a strong positive correlation (*r* > 0.5, *p* < 0.001) were considered to be *TET1*-coactivated genes. Correlation between gene expression and promoter methylation was calculated using Spearman’s method. The Mann–Whitney U test was used for between-group comparisons (e.g., PCa versus NOR, *TET1*-high versus *TET1*-low PCa) of *TET1* methylation at 30 CpG sites. The Kruskal–Wallis test was used to compare *TET1* expression and *TET1* methylation at 30 CpG sites among different PCa stages (T2a–c, T3a–b, and T4) and Gleason grades (GS6, GS7, GS8, and GS ≥ 9). Statistical significance was adjusted using the Bonferroni method, and *p* values < 0.05 were considered significant.

## Results

### TET1-expressing cells and cell clusters are frequently encountered in PCa and seldom present in benign prostate tissues

**Fig. 1:**
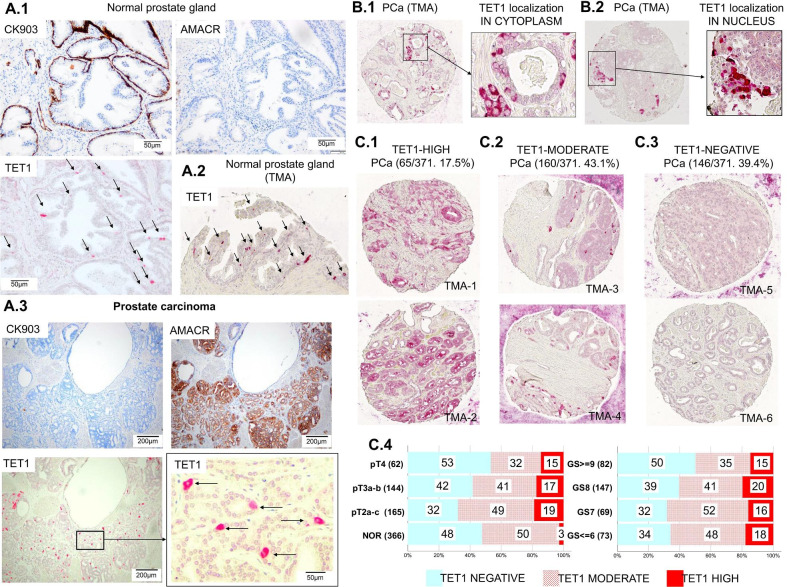
Analysis of TET1 expression by immunohistochemistry using conventional paraffin slides and tissue microarrays (TMA) in normal prostate (NOR) and prostate carcinoma tissue (PCa). **A** In NOR, TET1 was expressed in CK903-positive and AMACR-negative normal basal epithelial cells; these TET1-positive cells were rare and scattered in the basal epithelium (**A.1** and **A.2**). In PCa, TET1 was expressed in CK903-negative and AMACR-positive cancer cells, and TET1-expressing cells appeared much more frequently than in NOR (**A.3**); **B** TET1 expression was detected in both the cytoplasm (**B.1**) and the nucleus (**B.2**); **C** single NOR- and PCa-TMA spots were categorized according to the TET1 expression level as TET1-high (**C.1**, e.g., TMA-1 and TMA-2), TET1-moderate (**C.2**, e.g., TMA-3 and TMA-4), or TET1-negative (**C.3**, e.g., TMA-5 and TMA-6). The frequencies of TET1-high, moderate, and low TMA spots were calculated among NOR in comparison with PCa in regard to tumor stage (T2 to T4) and Gleason score (GS6 to GS9) (**C.4**)

Using conventional tissue slides, we examined TET1 expression by IHC in NOR and PCa specimens obtained from 50 PCa patients (Table 1, Fig. 1A). Particular regard was given to the cell specificity of TET1 expression. The basal cell marker CK903 and PCa cell marker AMACR were examined in parallel to TET1. In NOR, TET1 was exclusively expressed in CK903-positive basal cells, and TET1-expressing cells were scattered in the basal epithelium (Fig. 1A.1 and Additional file 1: Fig. S1A). In contrast, in PCa, TET1 was abundantly expressed exclusively in AMACR-positive cancer cells, whereby not all AMACR-positive cells expressed TET1 (Fig. 1A.3 and Additional file 1: Fig. S1C).

For a comprehensive overview, we performed a large-scale IHC study of TET1 expression using TMAs representing NOR and PCa specimens from 371 PCa patients (Table 2). In total, we could analyze 1371 single PCa-TMA spots and 669 single NOR-TMA spots. Observations in TMAs confirmed the results obtained from IHC analyses using conventional slides. In NOR-TMA, TET1 showed a scattered expression pattern in basal epithelial cells (Fig. 1A.2 and Additional file 1: Fig. S1B). In PCa-TMA, TET1-expressing cells appeared much more frequently and formed clusters (Fig. 1B, C.1, and Additional file 1: Fig. S1D). In both NOR and PCa, TET1 expression was detectable in the cytoplasm (Fig. 1B.1) as well as in the nucleus (Fig. 1B.2). Single PCa- and NOR-TMA spots could be categorized with respect to numbers of TET1-expressing cells as “TET1-high” (presence of numerous TET1-expressing cell clusters) (Fig. 1C.1), “TET1-moderate” (presence of a few scattered TET1-expressing cells) (Fig. 1C.2), or “TET1-negative” (absence of TET1-expressing cells) (Fig. 1C.3). From 371 PCa patients, 65 (17.5%) exhibited at least one PCa-TMA spot with high TET1 expression, 160 (43.1%) exhibited at least one PCa-TMA spot with moderate TET1 expression (Fig. 1C.1, C.2), and 146 (39.4%) exhibited only TET1-negative PCa-TMA spots (Fig. 1C.3). Among 366 PCa patients for which NOR-TMA were analyzed, ten (3%) exhibited at least one NOR-TMA spot with high TET1 expression, 181 (50%) at least one NOR-TMA spot with moderate TET1 expression, and 175 (48%) exhibited only TET1-negative NOR-TMA spots (Fig. 1C.4). Comparison of PCa patients considering the tumor stages (pT2 to pT4) and Gleason scores (GS ≤ 6 to ≥ 9) revealed that “TET1-high” foci were detectable in PCa at any tumor stage and Gleason grade (Fig. 1C.4). The frequencies of occurrence of TET1-high, moderate, and negative TMA spots were similar in PCa specimens from tumors of different stages and Gleason grades (Fig. 1C.4).

### Upregulation of TET1 in PCa is caused by aberrant DNA methylation in the *TET1* promoter, 5′-UTR, and gene body

Cancer-associated up- and downregulation of proteins is often caused by epigenetic dysregulation of the genes. To understand the epigenetic reasons for TET1 upregulation in PCa, we used TCGA, in particular the methylome and transcriptome data generated on 341 PCa and 35 NOR specimens (Additional file 2: Table S1).

**Fig. 2:**
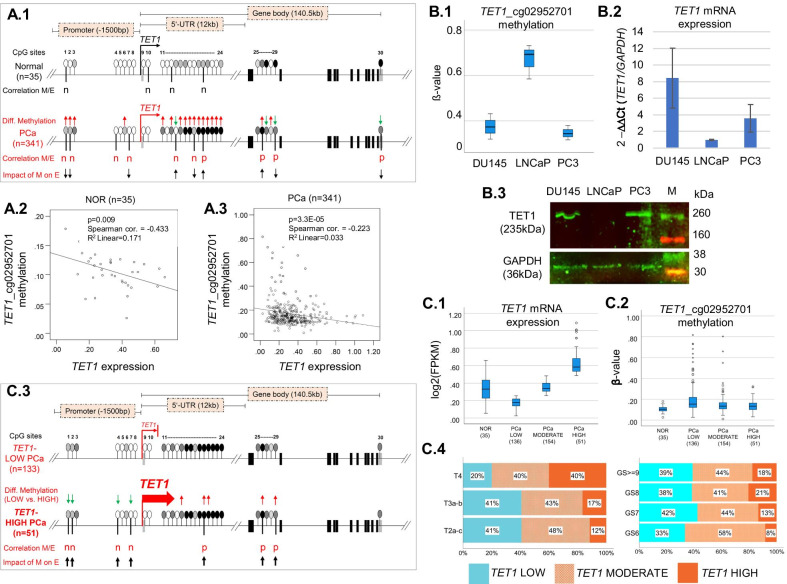
Analysis of *TET1* promoter methylation and expression in PCa and NOR tissue, and in PCa cell lines. **A** Based on the transcriptome data (FPKM: fragments per kilobase per million mapped reads) and methylome data (*ß*-values) of the Cancer Genome Atlas, we performed correlation analyses between *TET1* expression (E) and *TET1* methylation (M) at 30 CpG probes in 35 NOR and 341 PCa (A.1, Correlation of methylation with expression (M/E), n: significant negative correlation, p: significant positive correlation, Spearman). Comparison of *ß*-values at 30 CpG probes in PCa (341) and NOR (35) revealed 22 differentially methylated CpG sites in PCa (**A.1**, Diff. Methylation, 18 red up-arrows: 18 significantly hypermethylated sites in PCa, four green down-arrows: four significantly hypomethylated sites in PCa, Mann–Whitney U test). The impact of differential methylation at these 22 CpG sites on *TET1* expression was assessed according to the correlation of M/E in PCa (**A.1**, Impact of M on E, down arrows: downregulation, up arrows: upregulation). Methylation at *TET1*_cg02952701 showed the strongest correlation with *TET1* expression in both NOR (**A.2**) and PCa (**A.3**); **B** In LNCaP cells, *TET1*_cg02952701 was hypermethylated (**B.1**), and *TET1*-mRNA and TET1-protein were downregulated (**B.2** and **B.3**). In DU145 and PC3 cells, *TET1*_cg02952701 was hypomethylated (**B.1**), and *TET1* mRNA and TET1 protein were expressed (**B.2** and **B.3**); **C** based on *TET1* expression, we categorized the PCa cohort into three groups: low (expression < 40th percentile, *n* = 136), moderate (≥ 40th and ≤ 85th percentile, *n* = 154), and high (> 85th percentile, *n* = 51) (**C.1**). Accordingly, the *TET1*-high group showed the least methylation in *TET1*_cg02952701 and the *TET1*-low group showed the most methylation (**C.2**). Comparison of *ß*-values at 30 CpG probes in *TET1*-high PCa versus *TET1*-low PCa revealed nine differentially methylated CpG-sites in *TET1*-high PCa (**C.3**, Diff. Methylation, four green arrows: four significantly hypomethylated sites, five red arrows: five significantly hypermethylated sites). The four hypomethylated CpG sites in the promoter were significantly negatively correlated with *TET1* expression and thus, led to *TET1* upregulation. Three out of five hypermethylated CpG sites in the 5′-UTR and gene body were significantly positively correlated with *TET1* expression and thus also led to *TET1* upregulation (**C.3**, Correlation M/E, *n*: significant negative correlation, *p*: significant positive correlation, Spearman; Impact of M on E, up arrows: upregulation). Grouping of PCa by tumor stage and Gleason score revealed that *TET1*-high PCa occurred more frequently in advanced stages of PCa (**C.4**)

An initial comparison of *TET1* expression in PCa and NOR without any categorization of tissue samples showed no significant differences (Additional file 1: Fig. S2A). A comparison of PCa samples with regard to *TET1* expression in consideration of Gleason grade and tumor stage also showed no significant differences (Additional file 1: Fig. S2B). As methylation changes leading to aberrant gene expression may happen at specific sites throughout a gene, we aimed to systematically analyze all available *TET1* CpG probes in TCGA and to identify those critically important for *TET1* expression. Altogether, 30 CpG probes, including 8 in the promoter (TSS200 and TSS1500), 16 in the 5′-UTR, and 6 in the gene body of *TET1* were available in TCGA (Additional file 2: Table S2). Spearman’s analysis of possible correlation between *TET1* methylation at 30 CpG probes and *TET1* expression revealed the methylation at 4 CpG sites in NOR (one in the promoter and 3 in the 5′-UTR) and 10 CpG sites in PCa (4 in the promoter, 3 in the 5′-UTR, and 3 in the gene body) to be significantly correlated with *TET1* expression (Fig. 2A.1 and Additional file 2: Table S3). In NOR, methylation of all four CpG sites was negatively correlated with *TET1* expression (i.e., the more they were methylated, the less the gene was expressed). In contrast, in PCa, methylation of 6 out of 10 detected CpG sites was negatively correlated with *TET1* expression, and methylation of 4 was positively correlated (i.e., the more methylation, the more *TET1* was expressed) (Fig. 2A.1 and Additional file 2: Table S3). These data show the complexity of *TET1* dysregulation in PCa. Further differential methylation analyses of 30 *TET1* CpG probes in PCa versus NOR revealed 18 significantly hypermethylated CpG probes in PCa (4 in the promoter, 12 in the 5′-UTR, and 2 in the gene body) and 4 significantly hypomethylated CpG sites in PCa (one in the 5′-UTR and 3 in the gene body) (Fig. 2A.1, Additional file 2: Table S3 and Additional file 1: Fig. S3). Comparison of PCa specimens with regard to Gleason scores revealed 7 CpG sites in *TET1* (2 in the promoter and 5 in the gene body) to be significantly hypermethylated or hypomethylated in the course of prostate carcinogenesis (Additional file 1: Fig. S3).

Next, we selected the *TET1* CpG probe showing the most significant correlation with *TET1* expression in PCa and NOR, and analyzed it in PCa cell lines with regard to *TET1* mRNA and TET1 protein expression. In both PCa and NOR, *TET1*_cg02952701 located in the *TET1* promoter showed the strongest correlation with *TET1* expression (Fig. 2A.2, A.3, Additional file 2: Table S3). Analyses in PCa cell lines showed that hypermethylation of *TET1*_cg02952701 in LNCaP cells (Fig. 2B.1) was associated with downregulation of both mRNA and protein expression (Fig. 2B.2, B.3). In comparison, in DU145 and PC3 cells, *TET1*_cg02952701 was hypomethylated (Fig. 2B.1) and TET1 mRNA and protein were expressed (Fig. 2B.2, B.3). Thus, using PCa cell lines, direct causality between *TET1* promoter hypomethylation and upregulation of *TET1* mRNA and TET1 protein expression was confirmed.

Next, we categorized the TCGA-PCa specimens into three groups according to their *TET1* expression levels: *TET1*-high (expression level above the 85th percentile, *n* = 51), *TET1*-low (expression below the 40th percentile, *n* = 136), and *TET1*-moderate (expression between the 40th and 85th percentiles, *n* = 154) (Fig. 2C.1), and analyzed the methylation of *TET1*_cg02952701. As expected, hypomethylation of *TET1*_cg02952701 was observed in the *TET1*-high group, and hypermethylation in the *TET1*-low group (Fig. 2C.2). We then performed differential methylation analyses considering all 30 *TET1* CpG sites in *TET1*-high versus *TET1*-low PCa. In *TET1*-high PCa, four significantly hypomethylated CpG-sites in the *TET1*-promoter and five significantly hypermethylated CpG-sites in the *TET1* 5′-UTR and gene body were detected (Fig. 2C.3, Additional file 2: Table S4 and Additional file 1: Fig. S4). Importantly, the four hypomethylated CpG-sites were significantly negatively correlated with *TET1* expression, that is, they contributed to a gain of *TET1* expression, and the three hypermethylated CpG sites were significantly positively correlated with *TET1* expression, that is, they also contributed to a gain of *TET1* expression (Fig. 2C.3 and Additional file 2: Table S3). Thus, seven differentially methylated CpG-sites in *TET1* caused an overexpression of *TET1* in PCa. Further analyses showed that *TET1*-high PCa specimens were more frequently detected among PCa with higher Gleason scores (Gleason 6: 8.4%, Gleason 7: 13.3%, Gleason 8: 20.5%, Gleason ≥ 9: 17.6%) and higher tumor stages (T2a–c: 11.5%, T3a–b: 16.6%, T4: 40%) (Fig. 2C.4).

### Identification of 626 *TET1*-coactivated genes in PCa

**Fig. 3:**
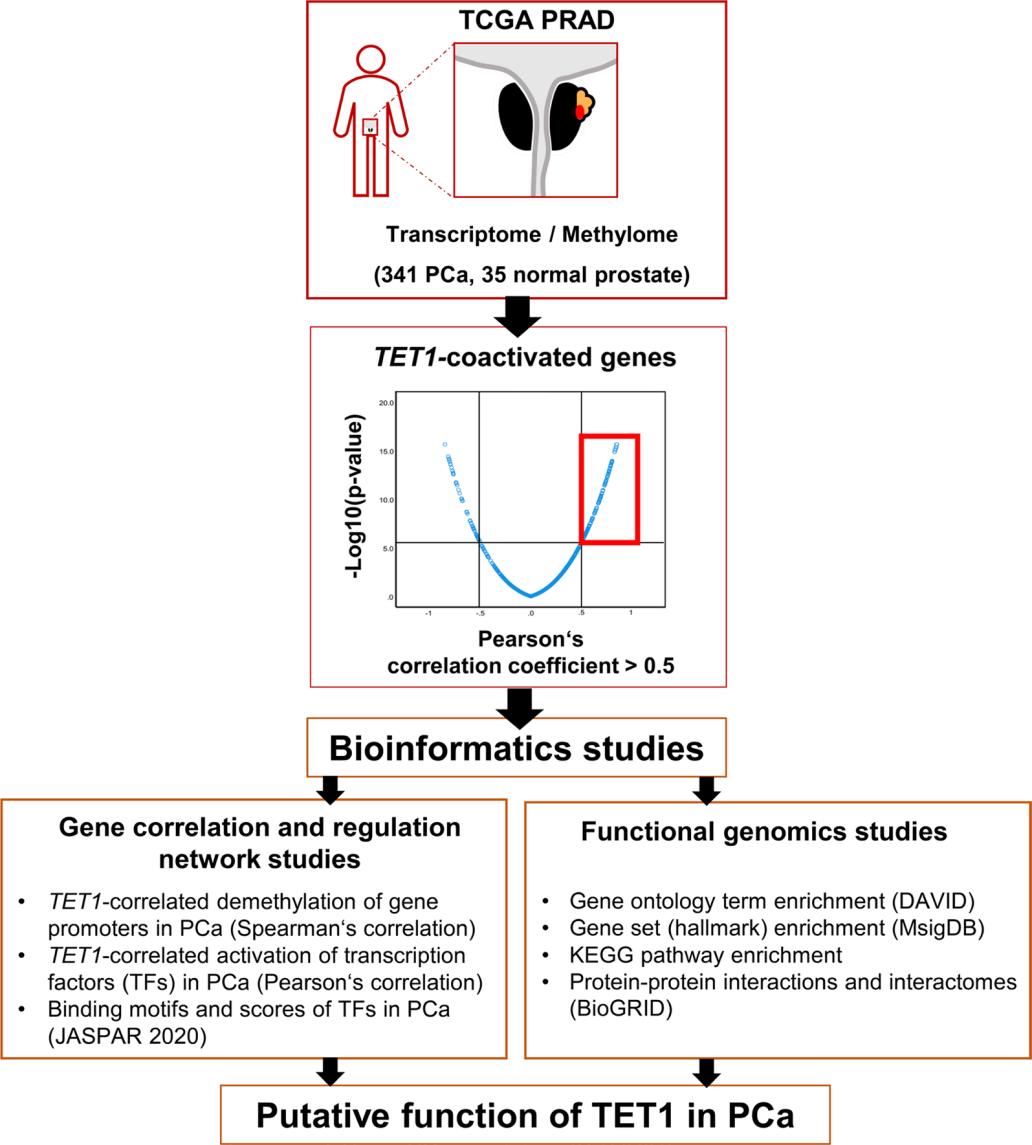
Work plan and methodology for creating a *TET1*-correlated gene regulation network and for functional genomics. The transcriptome and methylome data in the Cancer Genome Atlas (TCGA) generated from 341 PCa, were applied. *TET1*-coactivated genes were evaluated using Pearson’s correlation, and genes having *r* > 0.5 were considered in bioinformatics studies

To investigate the impact of cells and cell clusters in PCa expressing TET1 and *TET1* at high levels, we performed genome-wide correlation and gene regulatory network analyses using transcriptome and methylome data of PCa patients listed in TCGA (Fig. 3). Based on transcriptome data, we calculated the Pearson’s correlation coefficients between *TET1* expression and the expression values of all 19,252 protein-coding genes. Of 7533 genes exhibiting a statistically significant positive correlation to *TET1* expression (*p* < 0.05), 626 showed particularly strong correlation (Pearson’s *r* > 0.5, *p* < 0.0001). These 626 genes were referred to as *TET1*-coactivated genes and were subjected to further detailed bioinformatics studies (Fig. 3). *TET1* expression was not correlated to *AMACR* expression (Pearson’s *r* = 0.01, *p* = 0.8). As TET1 is a 5-methylcytosine dioxygenase, and demethylation of gene promoters leads to increased expression of the respective mRNA, we also calculated the Spearman’s correlation coefficients for the promoter methylation of 626 *TET1*-coactivated genes and *TET1* expression. Of 618 genes represented in the Illumina 450 K array, 594 possessed CpG sites in their promoters (sequences within 1500 and 200 bp of the transcriptional start site—TSS1500 and TSS200, respectively—were considered). In 279 out of 594 genes, the promoter was hypomethylated when *TET1* expression was increased, and in *TET1*-high PCa these gene promoters were significantly less methylated than in *TET1*-low PCa. In 235 of those 279 genes, promoter methylation was significantly negatively correlated with the gene’s expression, that is, the hypomethylation of the promoter led to increased gene expression.

### Upregulation of *TET1* in PCa strongly correlates with promoter demethylation and enhanced expression of genes encoding zinc-finger transcription factors

**Fig. 4:**
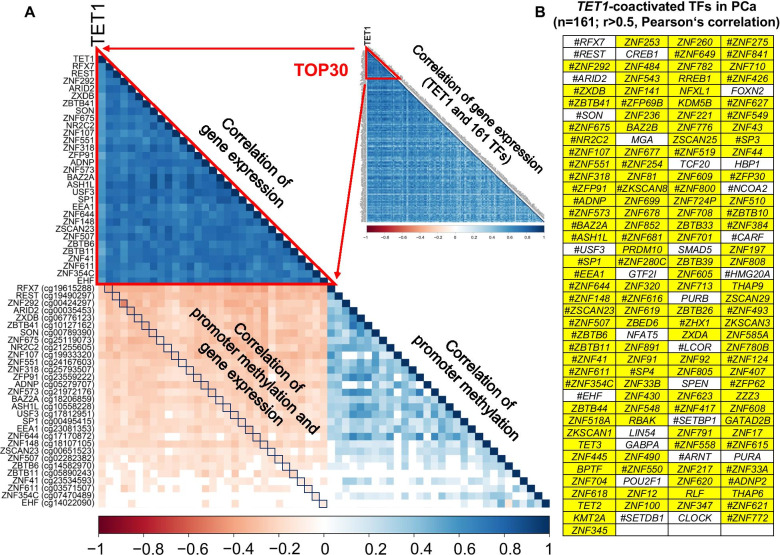
*TET1-*coactivated transcription factors (TFs) in PCa. **A** Correlation matrix (Pearson) demonstrating the strongly correlated expression of *TET1* and 161 genes encoding TFs in PCa. For TOP30 *TET1*-coactivated TFs (TF-encoding genes exhibiting the highest correlation with *TET1*), correlation matrices of gene expression (top triangle, Pearson), promoter methylation, and corresponding gene expression (black squares, Spearman; CpG probes in TSS1500 showing the most negative correlation with gene expression are indicated), and of promoter methylations (bottom triangle, Pearson; the methylation status of several promoters showed positive correlations) are shown; **B** 133 out of 161 *TET1*-coactivated TFs are zinc-finger TFs (highlighted in yellow), and in *TET1*-high PCa 68 showed at least one significantly demethylated CpG site within the TSS1500 promoter (hash mark; *TET1*-high versus *TET1*-low PCa, Benjamini–Hochberg method)

According to the Human Transcription Factor (TF) databank [30], 161 out of 626 *TET1*-coactivated genes in PCa are identified TFs. Remarkably, 133 out of 161 *TET1*-coactivated TFs (82.6%) belong to the group of zinc-finger TFs (Fig. 4) and were significantly enriched for the GO “Molecular function” terms “DNA-binding transcription activator activity” (23/133, *p* = 5.0e−08) and “DNA-binding transcription repressor activity” (15/133, *p* = 1.0e−07). Among the top 30 *TET1*-coactivated TFs, we found primarily stem cell- and cancer-associated TFs, for example, RFX7 (an X-box-recognizing TF involved in cellular specialization and differentiation), REST (a Kruppel-type TF involved in the regulation of stem cell pluripotency), ZNF292 (a growth hormone-dependent TF with tumor suppressor activity), ARID2 (an AT-rich interactive domain-containing TF involved in embryonic patterning and cell lineage gene regulation), ZXDB (an X-linked TF promoting MHC gene expression), NR2C2 (a nuclear hormone receptor involved in aging-associated diseases), and SP1 (a Kruppel-like TF involved in cell growth and differentiation) (Fig. 4A). Importantly, the genes encoding the top 30 *TET1*-coactivated TFs all possess at least one promoter CpG site that was significantly demethylated when *TET1* was significantly upregulated, and all these genes possessed significantly hypomethylated promoters in *TET1*-high PCa versus *TET1*-low PCa (Fig. 4B and Additional file 2: Table S6). In total, we identified 68 TFs in PCa whose gene promoters were significantly hypomethylated when *TET1* was significantly upregulated (Additional file 2: Table S6).

Next, using JASPAR 2020 we analyzed the binding motifs and binding scores of *TET1*-coactivated TFs in the *TET1* promoter and in the promoters of 626 *TET1*-coactivated genes (TSS1500 was considered). Of 161 TFs, 21 were listed in JASPAR 2020 and could be analyzed. In the *TET1* promoter, TF-BSs were found for all 21 analyzed TFs (Additional file 2: Table S5). Segments in the *TET1* promoter that were found to be significantly hypomethylated in *TET1*-high PCa and hence decisive for *TET1*-upregulation (Fig. 2C.3 and Additional file 2: Table S4) exhibited TF-BSs for RFX7, NR2C2, SP1, CREB1, MGA, SMAD5, ZBTB6, ZSCAN9, and ZNF354C (Additional file 2: Table S4). The vast majority of 626 *TET1*-coactivated genes also exhibited TF-BSs for 19 out of 21 TFs (Additional file 2: Table S5). The mean number of TF-BSs per gene promoter varied from a few (1 to 10), for example, in ZBTB26, ZKSCAN1, ZKSCAN29, SMAD5, POU2F1, ZBTB6, SP4, ZNF148, EHF, GABPA, CREB1, RREB1, and REST, to a great many (> 10), for example, in ZNF384, NR2C2, ZNF354C, RFX7, MGA, CLOCK, SP3, and SP1 (Additional file 2: Table S5). Nine out of 21 *TET1*-coactivated TFs, namely RFX7, SP1, SP3, SP4, POU2F1, ZBTB6, REST, CLOCK, and ZSCAN29, possessed CGs in their binding motifs and hence were sensitive to CpG-methylation and demethylation (Additional file 1: Fig. S5).

Many TFs of different families do not work alone and form complex homotypic or heterotypic interactions through dimerization [38]. Therefore, we analyzed the protein–protein interactions (PPI) and PPI networks (interactomes) of 161 *TET1*-coactivated TFs. Interactome analyses centered on 21 TFs that were available in JASPAR 2020 and showed true binding sites in the *TET1* promoter and promoters of *TET1*-coactivated genes. Two interactomes, one SP1-centered and one CREB1-centered, were revealed (Additional file 1: Fig. S6). SP1, a zinc-finger transcription activator binding to GC-rich motifs, showed a direct interaction with six TFs (POU2F1, GABPA, ARNT, SP3, REST, and PURA) (Additional file 1: Fig. S6A.1) and an indirect interaction (i.e., through a bridge TF) with another 33 TFs (Additional file 1: Fig. S6A.2). CREB1, a transcription activator that binds to the cAMP response elements in many mammalian and viral promoters, showed a direct interaction with three TFs (ZHX1, POU2F1, and ZNF92) (Additional file 1: Fig. S6B.1) and an indirect interaction with a further 31 TFs (Additional file 1: Fig. S6B.2).

### *TET1*-coactivated genes in PCa point to a cumulative gain of chromatin remodeling and mitotic activities

**Fig. 5:**
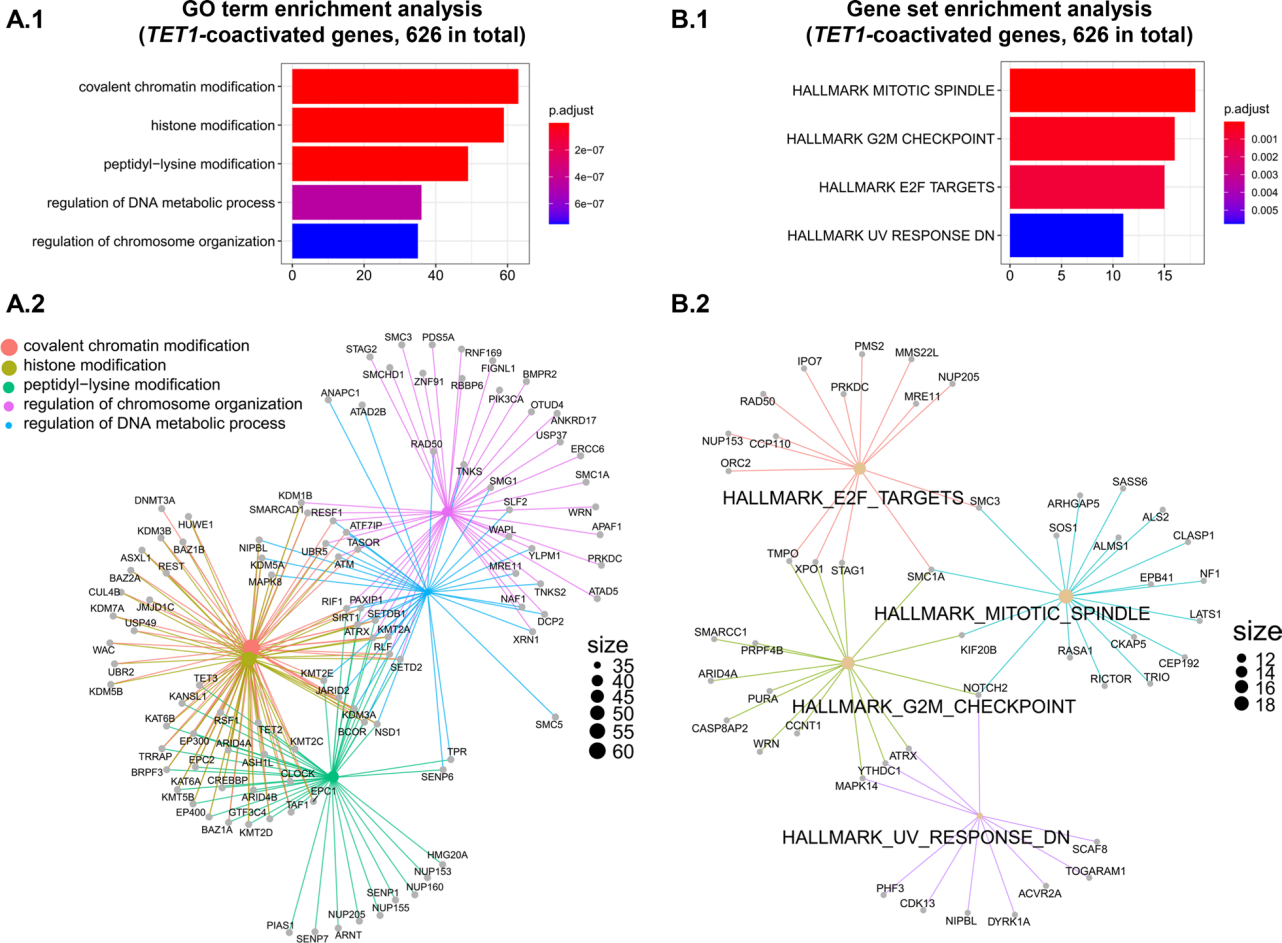
Functional characterization of *TET1*-coactivated genes in PCa. **A** Gene ontology (GO) term enrichment analysis of 626 *TET1*-coactivated genes (**A.1**) and network plot of enriched terms depicting the linkages of individual genes and GO terms (**A.2**) (adjusted *p* values and numbers of included genes are given); **B** gene set (hallmark) enrichment analysis of 626 *TET1*-coactivated genes (**B.1**) and network plot of enriched terms depicting the linkages of individual genes and hallmarks (**B.2**) (adjusted *p* values and numbers of included genes are given)

To characterize the functional features shared by 626 *TET1-*coactivated genes in PCa, we performed functional genomics studies (Fig. 3). GO analyses revealed a significant enrichment of biological processes primarily responsible for chromatin remodeling, such as “covalent chromatin modification,” “histone modification,” and “peptidyl-lysine modification” (Fig. 5A and Additional file 2: Table S7). In total, 78 genes encoding epigenetic modifiers were activated in PCa together with *TET1* (Additional file 2: Table S7 and Additional file 1: Fig. S7). Moreover, significant enrichments of biological processes involved in regulating DNA metabolism and chromosome organization were also detected (Fig. 5A). Among *TET1*-coactivated genes, we found *TET2*, *TET3,* and *DNMT3A*. In both NOR and PCa, strong positive correlations between the expression of *TET1, TET2, TET3,* and *DNMT1* were found (Additional file 1: Fig. S8). However, only in PCa, strong positive correlations were also found between *TET1* and *DNMT3A* and *DNMT3B* expression (Additional file 1: Fig. S8). Further, GSEA of “Hallmark gene sets” on 626 *TET1*-coactivated genes showed a significant enrichment of mitotic and DNA replication hallmarks, in particular “mitotic spindle,” “G2M checkpoint,” and “E2F targets” (Fig. 5B and Additional file 2: Table S7). In total, 41 genes encoding mitotic factors were activated in PCa, together with *TET1* (Additional file 2: Table S7). In order to examine, whether the P53-pathway is affected alongside with the E2F targets, we performed a GSEA of “Curated gene sets,” in particular gene sets representing canonical pathways. Among 626 *TET1*-coactivated genes, we found significant enrichments for pathways related to regulation of P53 activity by phosphorylation and methylation, and to P53-hypoxia (Additional file 2: Table S7). Moreover, GSEA of “Oncogenic signature gene sets” representing cellular pathways often dysregulated in cancer [35] on 626 *TET1*-coactivated genes showed highly significantly enrichments for gene sets related to Kirsten rat sarcoma viral oncogene homolog (KRAS) and TANK Binding Kinase 1 (TBK1), Placental Growth Factor (PGF), Janus Kinase 2 (JAK2), Catenin Beta 1 (CTNNB1) and Vascular Endothelial Growth Factor A (VEGFA) (Additional file 1: Fig. S9 and Additional file 2: Table S8).

**Fig. 6:**
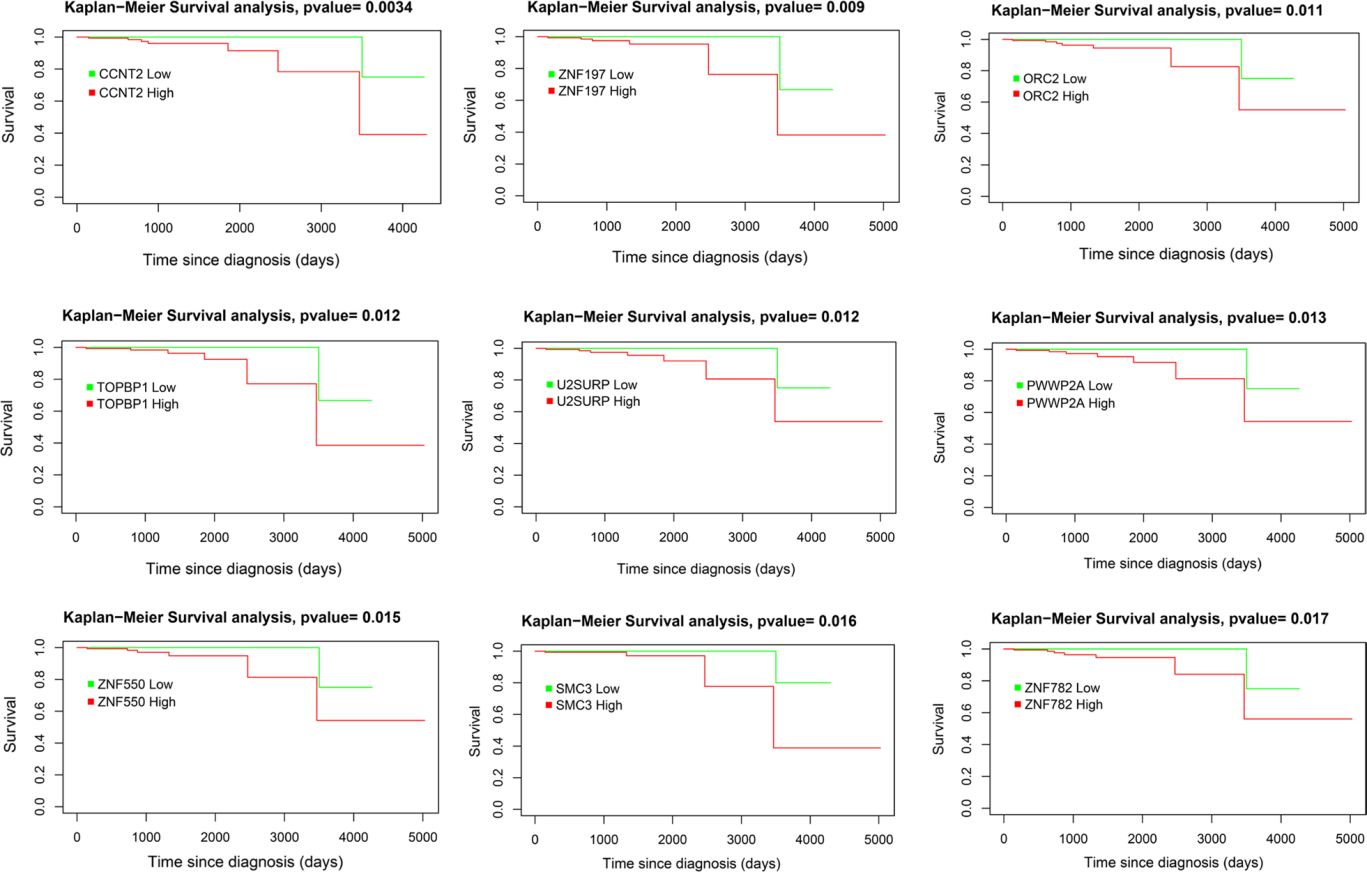
Kaplan–Meier survival analysis on *TET1*-coactivated genes in PCa. In total, expression of 35 out of 626 *TET1*-coactivated genes was significantly associated with survival. Survival curves of top 9 genes are shown (*p* value < 0.02)

Next, we performed TCGAanalyze_SurvivalKM, a univariate Kaplan–Meier (KM) survival analysis on 626 *TET1*-coactivated genes in PCa (Fig. 6, Additional file 2: Table S9). In total, expression of 35 genes showed a significant impact on survival (Additional file 2: Table S9). Survival curves of top 9 candidates (*CCNT2, ZNF197, ORC2, TOPBP1, U2SURP, PWWP2A, ZNF550, SMC3* and *ZNF782*) are shown in Fig. 6 (*p* value < 0.02).

**Fig. 7:**
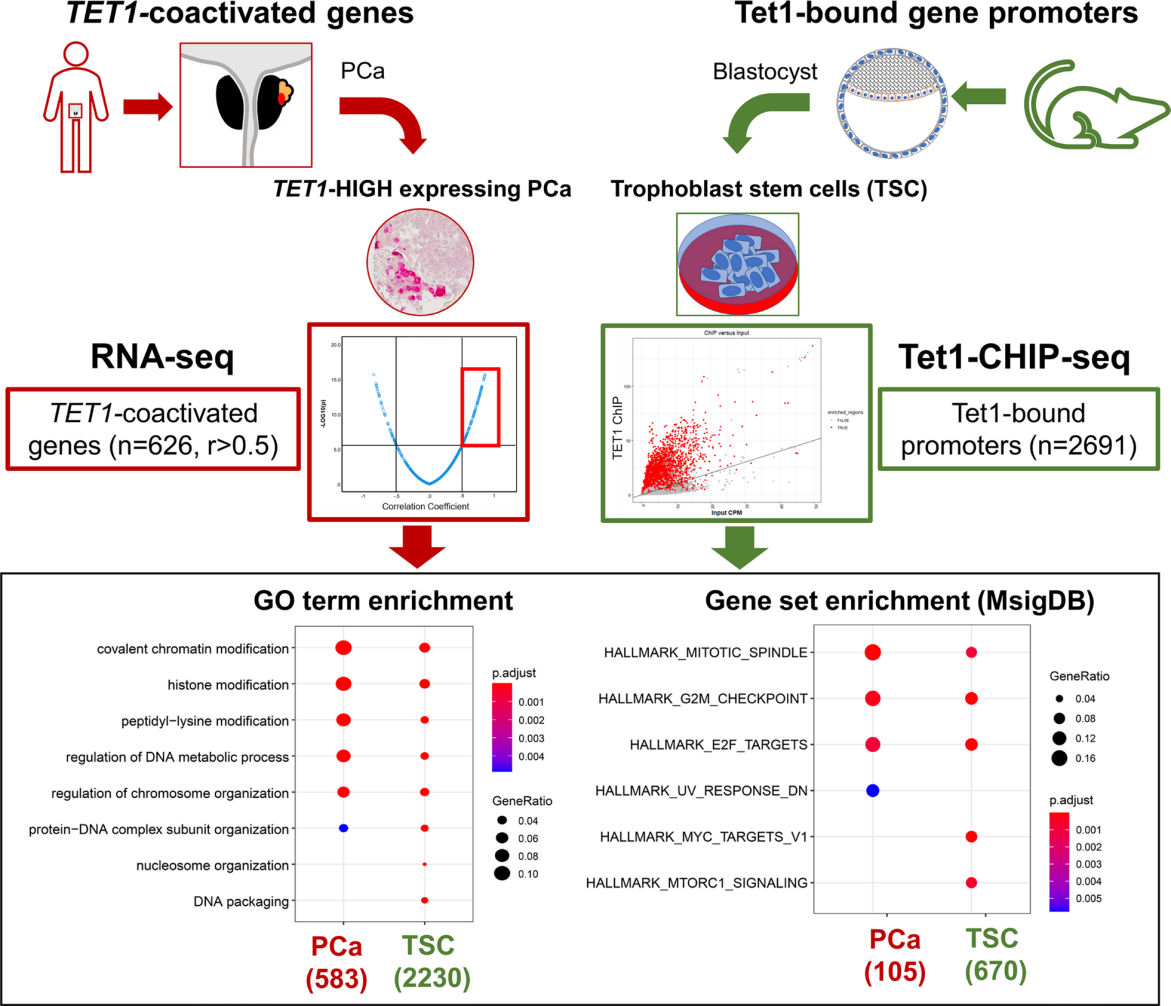
Comparison of *TET*-coactivated genes in PCa and Tet1-bound genes in mouse trophoblast stem cells. *TET1*-coactivated genes in PCa (626) were analyzed in parallel to genes exhibiting Tet1-binding sites within the TSS1500-promoter (2691) with regard to gene ontology (GO) term enrichment (bottom left), and gene set enrichment (bottom right; MsigDB: Molecular Signatures Database). The results showed enrichment of nearly exactly the same GO terms (primarily associated with chromatin modification) and gene sets (primarily associated with mitotic division) in the two groups

Analyzing publicly available TET1- and Tet1-ChIP-seq data, we discovered a strong similarity of MSigDB-hallmark- and GO-enrichment profiles in PCa and mouse trophoblast stem cells (mTSCs) [9, 10]. In mTSCs, 2691 gene promoters (defined as TSS1500) exhibited Tet1 binding sites (Fig. 7). A parallel analysis of *TET1*-coactivated genes in PCa and genes exhibiting Tet1 binding sites in mTSCs revealed an enrichment of nearly the same GO terms (primarily associated with chromatin modification) and same gene sets (primarily associated with mitosis) (Fig. 7).

### *TET1*-correlated activation of ZNF antiviral genes in PCa

To explore the pathways activated in *TET1*-high PCa, we performed KEGG pathway analyses on 626 *TET1*-coactivated genes (Additional file 1: Fig. S10). We found that the “herpes simplex virus 1 infection” (HSV-1 infection) pathway was the most significantly enriched one (Additional file 1: Fig. S10A and S10B). In total, 62 genes encoding ZNF antiviral proteins (ZAPs) showed a strong positive correlation to *TET1* expression and were significantly upregulated in *TET1*-high PCa (Additional file 1: Fig. S10C and Additional file 2: Table S10). Besides the genes encoding ZAPs, we also detected five genes encoding signaling molecules involved in the immune response to cancer, namely *TRAF6*, *EIF2AK2*, *PIK3CA, APAF1,* and POU2F2 (Additional file 1: Fig. S10C). Therefore, to assess the impact of viral infections in PCa, we analyzed 34 PCa and 16 benign prostate hyperplasia (BPH) samples with regard to different viral infections, including the herpesviruses HCMV, EBV and HSV-1/-2, and the polyomaviruses BK and JC (Additional file 2: Table S11). We detected infections with HCMV in 11/34 (32.4%) PCa and 3/16 (18.8%) BPH, and EBV in 5/34 (14.7%) PCa and 1/16 (6.3%) BPH (Additional file 2: Table S11). No infections with HSV-1/-2 or JC-/BK-viruses were found in PCa and BPH.

Furthermore, KEGG pathway analyses also revealed a significant enrichment of genes involved in “signaling pathway regulating pluripotency of stem cells” (Additional file 1: Fig. S10D and Additional file 2: Table S10). The expression of several genes encoding regulators of stem cell pluripotency, including *PI3K*, *ACVR1/2*, *BMPR1/2*, *GSK3B*, *p38*, *SMARCAD1*, *SETDB1*, *JARID2*, *KAT6A*, *REST,* and *RIF1* showed a strong positive correlation with *TET1* expression in PCa and were coactivated in *TET1*-high PCa (Additional file 1: Fig. S10D).

## Discussion

Cancer cells, also within a tumor, may be heterogenous and very with regard to their molecular profiles and strategies to achieve a growth advantage. The identification of self-renewing cancer stem cells driving tumor growth and metastasis is the primary objective of attempts to discover biomarkers in aggressive PCa. As a key regulator of stem cell pluripotency, and being dysregulated in a wide range of human malignancies, TET1 is a highly relevant candidate. As yet, TET1 has been poorly investigated in PCa. Prior studies on TET1 have analyzed a relatively small number of primary PCa specimens [28, 29, 39] or have been limited to cell culture experiments [40, 41]. TET1 has been shown to suppress PCa invasion by activating tissue inhibitors of metalloproteinases [28]. TET1 protein levels are decreased in PCa, and low *TET1* mRNA levels are significantly associated with worse metastasis-free survival [29]. However, distinct activating and repressive functions of TET1-mediated transcriptional regulation could also be demonstrated in PCa [39].

In this context, we performed a large-scale IHC study, analyzing a large number of tissue samples by IHC using both conventional slides (NOR and PCa specimens originating from 50 PCa patients) and TMA (669 single NOR-TMA spots and 1371 single PCa-TMa spots originating from 371 PCa patients). These two complementary methods allowed us to get a comprehensive and detailed view on TET1 expression in the prostate. Our data show that in normal prostate, TET1 was exclusively expressed in CK903-positive basal cells, which were scattered in the basal epithelium known to contain the prostate stem cells [12, 15]. In contrast, in PCa, where the basal cell layer was often destroyed, we could frequently detect TET1 expression in AMACR-positive cancer cells. Remarkably, TET1-expressing PCa cells often occurred in big clusters, and these clusters were observable at all tumor stages. However, among NOR-TMA spots, some few foci also possessed TET1-high-expressing cells, but the frequency of occurrence was sixfold lower than among PCa-TMA spots. Based on their TET1-expression profiles, PCa specimens could be stratified into three subgroups (TET1-high, moderate, and negative), and TET1-high PCa specimens exhibiting numerous clearly defined TET1-positive cell clusters accounted for 17.5% of all PCa. Regulation of DNA demethylation and, hence, the transcriptional activity of pluripotency genes is the key function of TET methyl-cytosine dioxygenases including TET1, and is essential for the maintenance of stem cell self-renewal capacity, as shown in various mouse and human stem cell types [42]. According to our results, in the normal prostate TET1 may be involved in maintaining basal cell stemness, and in PCa (more precisely in TET1-expressing cell clusters/colonies), it may be aberrantly upregulated and enforce oncogenic properties. Much evidence indicates that PCa arises from pluripotent CSCs that possess a certain level of plasticity and are major determinants of tumor development and progression [15]. The multitude of CSC surface and intracellular markers detected in PCa, for example, c-kit, CD133, CD44, α2β1 integrin, CXCR4, Sox2, Oct3/4, Nanog, c-myc, and Klf4 [15], emphasizes the molecular and biological diversity of CSCs in PCa. Our results suggest that TET1 expression could be characteristic of proliferating CSCs in PCa.

In our study, IHC revealed that TET1 may reside in the nucleus as well as in the cytoplasm in both PCa and the normal basal epithelium. Similar subcellular localization of TET1 has been observed in ovarian endometriotic lesions, gastric and ovarian cancers, hippocampal neurons, and gliomas [25, 43–46]. Some studies have addressed the impact of the nuclear/cytoplasmic TET1 ratio on genome-wide DNA methylation levels, but the results have been inconclusive. In cell culture, under low oxygen conditions, an observed increase in the nuclear/cytoplasmic TET1 ratio has been suggested to lead to an increase in 5hmC [10]. However, complete cytoplasmic TET1 translocation did not lead to significant alteration of 5hmC-levels [47]. O-GlcNAc transferase has been reported to physically and functionally interact with TETs and be responsible for their subcellular localization [48].

Next, by comparing *TET1*-high and -low PCa, we revealed in *TET1*-high specimens significantly demethylated CpG-sites in *TET1* promoter, which significantly correlated with an increased *TET1*-mRNA and TET1-protein expression. Remarkably, *TET1*-high PCa were more frequently detected among advanced tumor stages. In order to characterize the molecular profile of *TET1*-high PCa, we evaluated the expression of other genes for correlation with *TET1* expression. The correlation threshold set at 0.5 (Pearson’s *r* > 0.5) provided a conclusive balance between strong correlation and a sufficiently informative number of *TET1*-correlated genes. Among 626 genes upregulated together with *TET1* in PCa, significant enrichments were found primarily for genes encoding chromatin remodeling and mitotic factors. Furthermore, specific oncogenic signature gene sets and pathways were enriched too. Among *TET1*-coactivated genes, we also detected 161 encoding TFs, with a particular dominance of ZNF-TFs (133/161). All 21 TFs that were analyzed in detail had significant binding sites in the *TET1* promoter as well as in the promoters of the vast majority of *TET1*-coactivated genes. It is very likely that all 626 *TET1*-coactivated genes possess TET1-BSs in their promoters, as exactly the same GO terms, and gene hallmarks were enriched when Tet1-binding promoters in mouse TSCs were analyzed [9], and many exhibited significantly hypomethylated promoters in *TET1*-high in comparison with *TET1*-low PCa. These findings point to an orchestrated activation of *TET1* and these 626 genes, and together with our IHC observations strengthen the hypothesis that AMACR+/TET1+ cancer cells and cell clusters in PCa may constitute proliferating cell colonies and represent a specific cell entity with stem cell attributes and an oncogenic signature. There are a number of reports demonstrating the oncogenic potential of TET1, in particular in ovarian and breast cancer [49–51]. In triple-negative breast cancer, which is one of the most hypomethylated cancers, TET1-mediated hypomethylation activates oncogenic signaling [27]. The authors suggested that TET1, as a potential oncogene, could serve as a druggable target for therapeutic intervention [27]. Our results support this idea and furthermore suggest that TET1 might be a good candidate for tracing mitotically active cells with stem cell attributes in PCa.

It is known that the prostate normally contains abundant intracellular zinc and that dysregulation of zinc homeostasis, in particular zinc loss, is a hallmark of PCa development [52]. Interestingly, in addition to 133 ZNF-TFs, we found 62 ZAPs and several signaling molecules involved in the antiviral immune response to be activated at gene level together with *TET1* and enriched for the KEGG-pathway “HSV-1 infection.” Our findings emphasize the impact of zinc-dependent proteins and their dysregulation in PCa, and indicate a possible herpesvirus-mediated path of TET1 activation. However, we did not detect HSV-1/2-infections, but found twice as many infections with other herpesviruses HCMV and EBV in PCa as in BPH. Reactivation of HCMV may occur in previously immunocompetent patients post-operatively after chemo- or radiotherapy, and is associated with poor outcomes, including other infections and mortality [53]. Zinc is an essential trace element crucial for immune function, can influence antiviral immunity, and is discussed as possibly favorable additive against viral infections such as HSV and the common cold [54]. Thus, zinc-mediated therapeutics may also be an effective approach to PCa prevention and treatment.

In conclusion, our results suggest that in PCa, TET1-expressing cells and cell colonies may be proliferating tumor cells with a distinct oncogenic signature. Our study contributes to a better understanding of TET1 function in human malignancies and underlines its potential for marker development.

## Supplementary Information

**Additional file 1.** Supplementary Figures.

**Additional file 2.** Supplementary Tables.

## Data Availability

Not applicable.

## Abbreviations

TET1: Ten–eleven translocation family member 1
PCa: Prostate cancer
BPH: Benign prostate hyperplasia
NOR: Normal prostate
CSCs: Cancer stem cells
TMA: Tissue microarray
CK903: Cytokeratin 903
AMACR: Alpha-methylacyl-CoA racemase
ZNF: Zinc finger
ZAP: Zinc finger antiviral protein
TF: Transcription factor
TCGA: The Cancer Genome Atlas
DAVID: Database for Annotation, Visualization and Integrated Discovery
MSigDB: Molecular Signatures Database
GSEA: Gene set enrichment analysis
KEGG: Kyoto Encyclopedia of Genes and Genomes
PPI: Protein–protein interaction
HCMV: Human cytomegalovirus
EBV: Epstein–Barr virus
HSV: Herpes simplex virus
RT-qPCR: Reverse transcription quantitative polymerase chain reaction
