# Supplementary material for: Tracing TET1 expression in prostate cancer: discovery of malignant cells with a distinct oncogenic signature
Source: Clin Epigenetics. 2021 Nov 29;13:211. doi: 10.1186/s13148-021-01201-7 (PMC8630881; doi:10.1186/s13148-021-01201-7)
Supplement: Supplementary file 1 — Additional file 1. Supplementary Figures. [file 13148_2021_1201_MOESM1_ESM.pdf]

Supplementary Figures

Supplementary Figure 1) Immunohistochemical (IHC) analyses in normal prostate and prostate cancer. Ten Eleven Translocation family protein 1 (TET1), basal cell marker cytokeratin 34 beta E12 (also CK903) and tumor cell marker Alpha-Methylacyl-CoA-Racemase (AMACR) were analyzed by IHC. **A and B)** In normal prostate, TET1-protein expression was detectable only in few scattered basal epithelial cells (marked with arrows), within the CK903-positive basal cell layer (TMA: tissue microarray); **C and D)** In PCa, TET1-protein expression was frequently detectable in AMACR-positive tumor cells, but not in CK903-positive basal cells as shown in serial sections of a paraffin-embedded PCa tissue (C), and TET1-expressing cells appeared often in big cell clusters as shown in different TMA-spots (D).

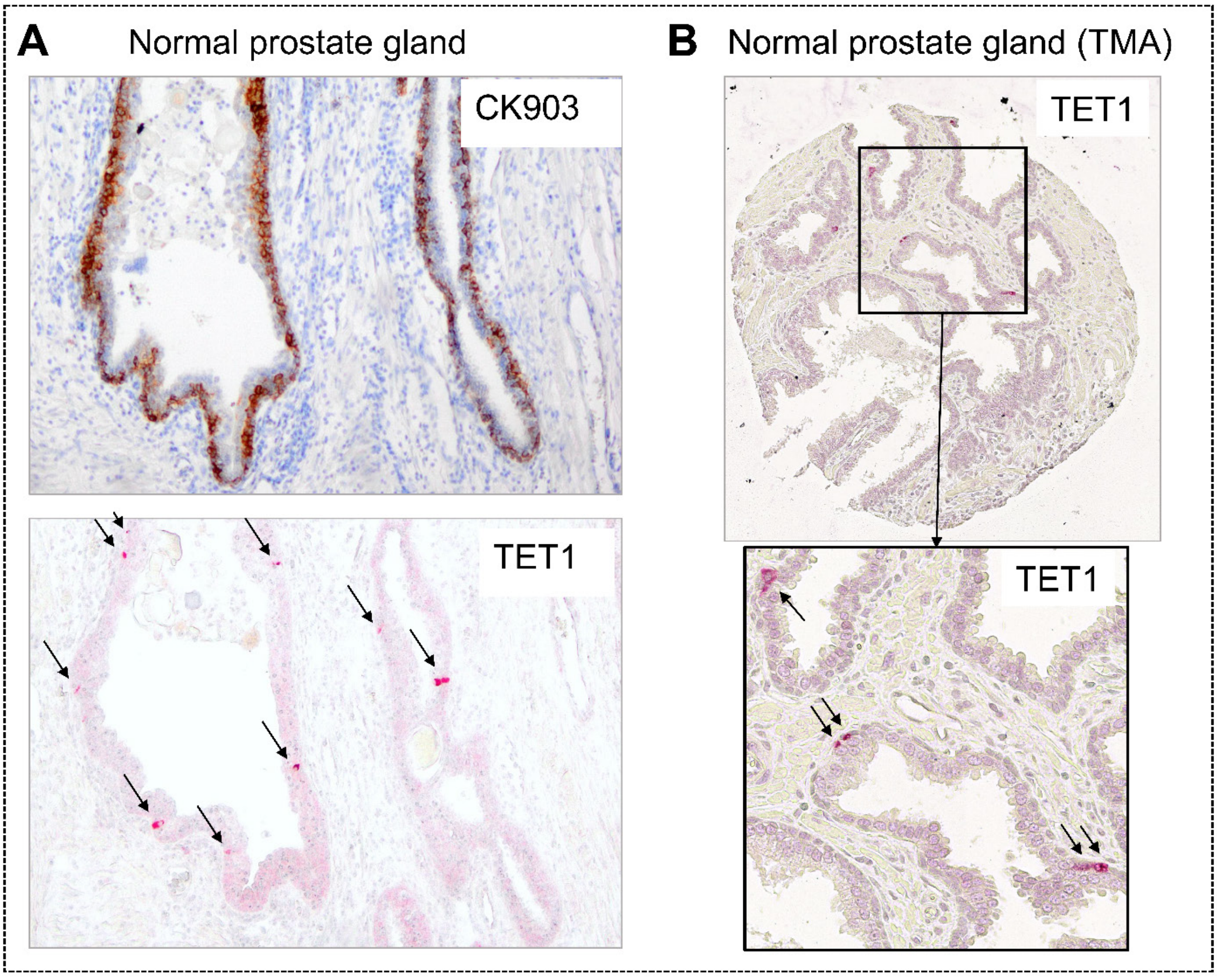

C Prostate carcinoma (co expression analysis)

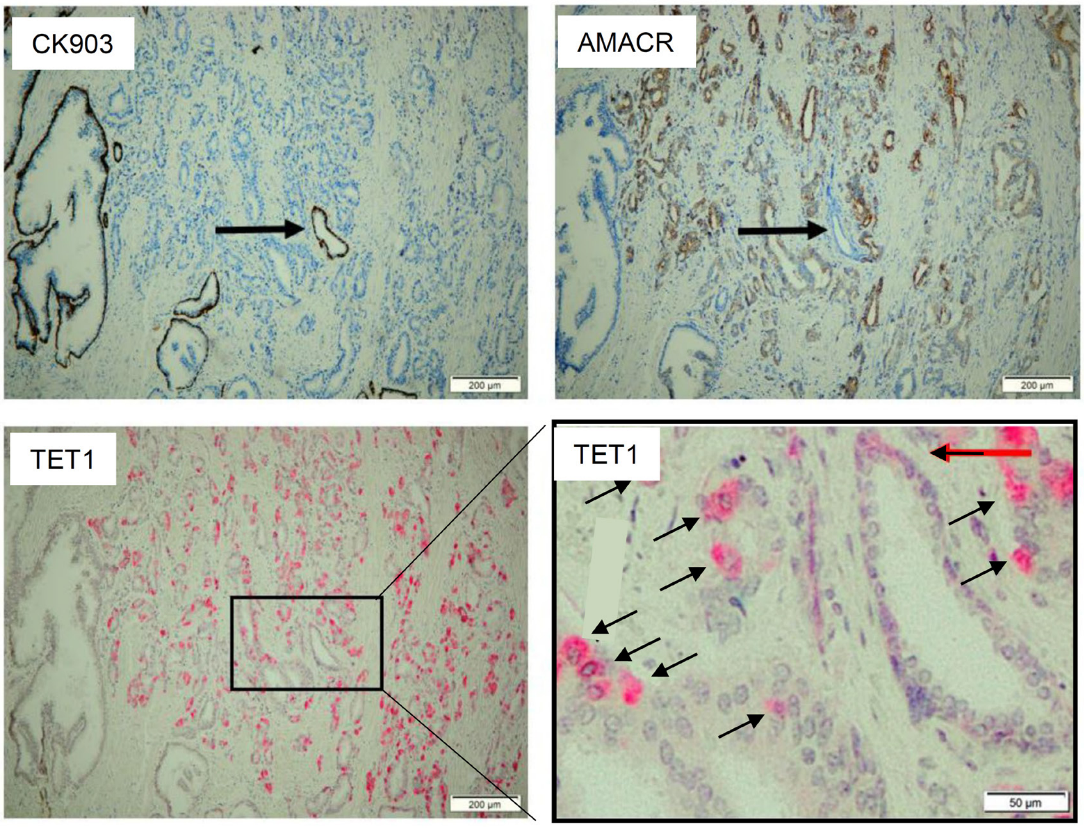

D Prostate carcinoma 1 (TMA)

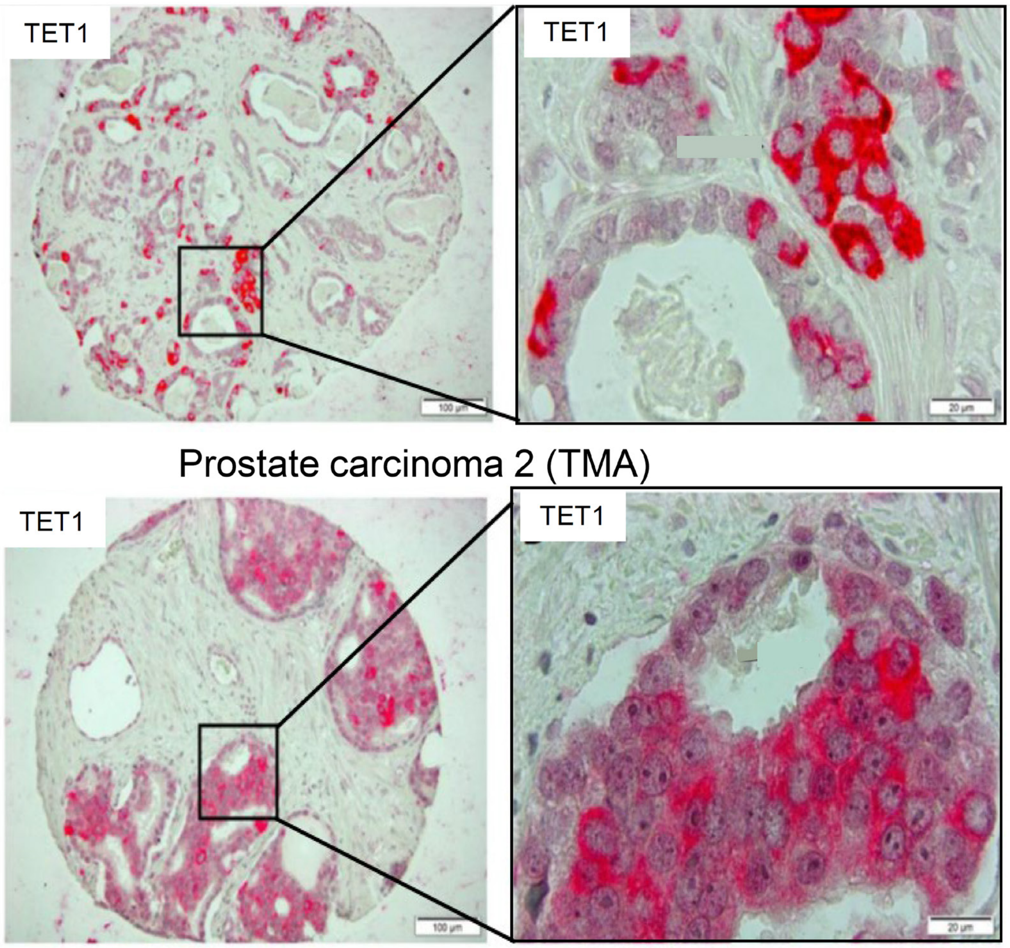

Supplementary Figure 2) Gene expression analyses of *TET1* in normal prostate and prostate cancer. A) Using The Cancer Genome Atlas (TCGA) database, we compared the expression of *TET1* gene in normal prostate (NOR, n=35) and prostate cancer (n=341), and no significant differences were detected (Mann-Whitney-U test); **B)** Expression of *TET1* gene was also analyzed among tumors with different Gleason scores (GS6 n=51, GS7 n=118, GS8 n=29, GS9 n=31) and tumor sizes (T2a-c n=130, T3a-b n=199, T4 n=5), and here, too, no significant differences were detected (Kruskal-Wallis-test).

A

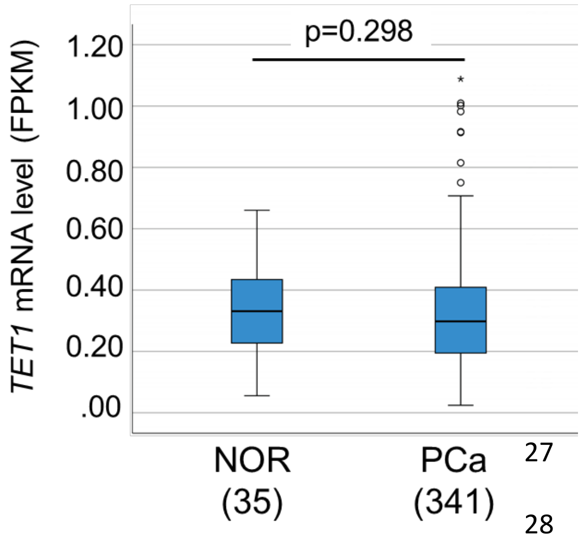

B

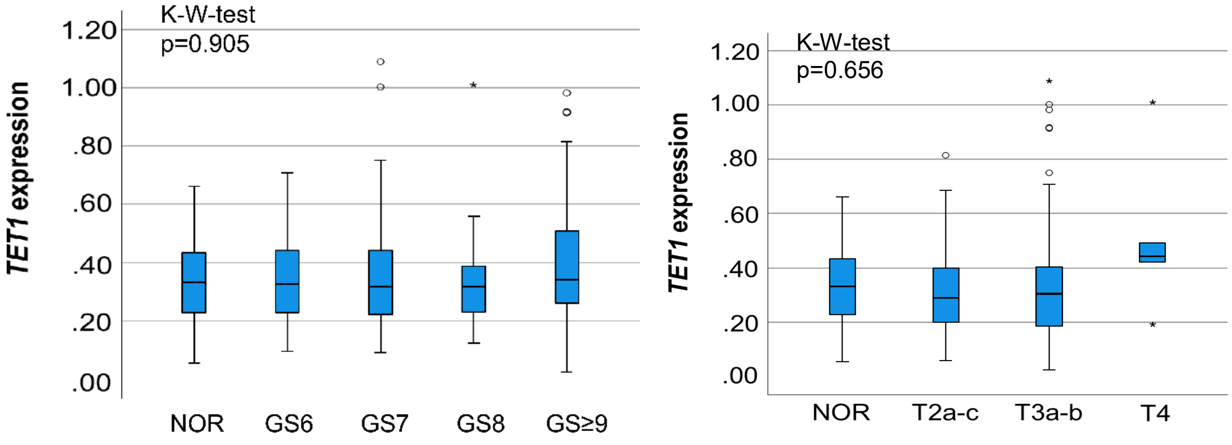

Supplementary Figure 3) Differential methylation analyses in *TET1* gene. 30 CpG-probes in *TET1* promoter, 5'-UTR and gene body were analyzed using TCGA database in 35 normal prostate (NOR) and 341 prostate cancer samples with different Gleason scores (GS6 n=51, GS7 n=118, GS8 n=29, GS9 n=31) (between-group-comparisons were done using Kruskal-Wallis-test, p-values are indicated; cg-IDs correspond to TCGA database, CpG-locations in *TET1* correspond to STable 2). 22 out of 30 CpG-probes were differentially methylated in PCa in comparison to NOR (s. also STable 2), and 7 out of 30 CpG-probes were differentially methylated among different GSs (in Promoter/1/5 and Body/25/27/29/30).

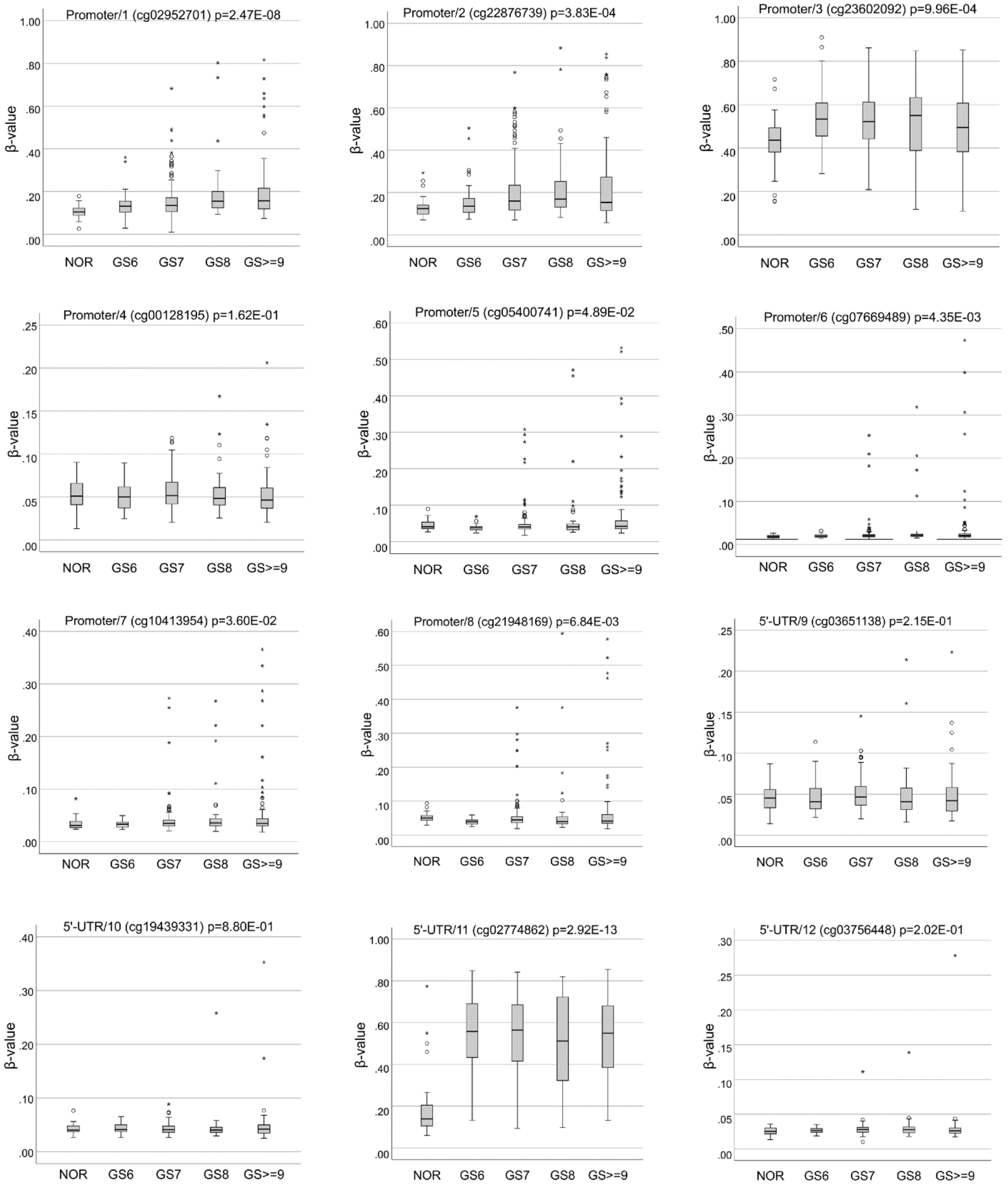

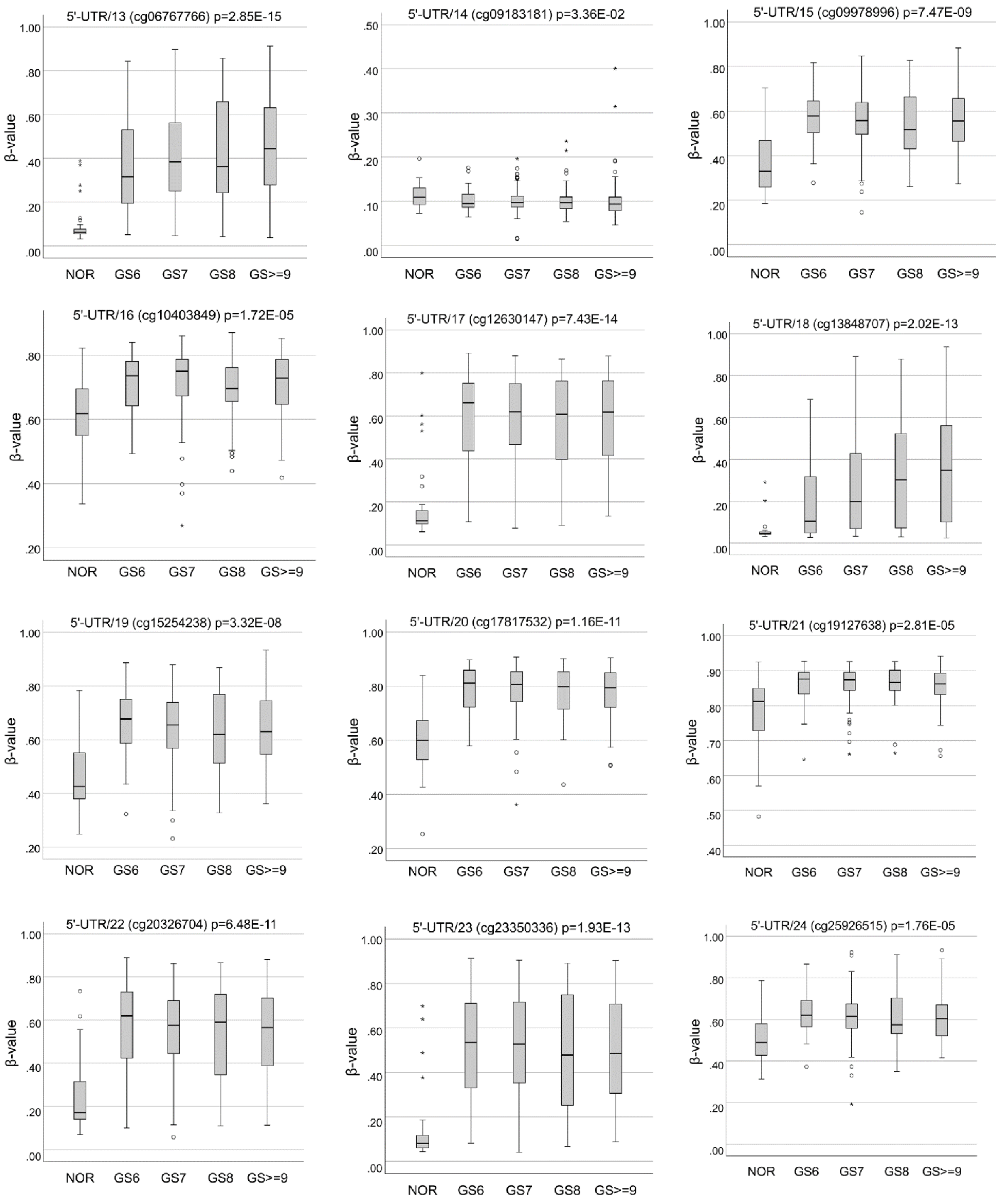

5'-UTR/13 (cg06767766) p=2.85E-15
5'-UTR/14 (cg09183181) p=3.36E-02
5'-UTR/15 (cg09978996) p=7.47E-09
5'-UTR/16 (cg10403849) p=1.72E-05
5'-UTR/17 (cg12630147) p=7.43E-14
5'-UTR/18 (cg13848707) p=2.02E-13
5'-UTR/19 (cg15254238) p=3.32E-08
5'-UTR/20 (cg17817532) p=1.16E-11
5'-UTR/21 (cg19127638) p=2.81E-05
5'-UTR/22 (cg20326704) p=6.48E-11
5'-UTR/23 (cg23350336) p=1.93E-13
5'-UTR/24 (cg25926515) p=1.76E-05
β-value
NOR
GS6
GS7
GS8
GS>=9

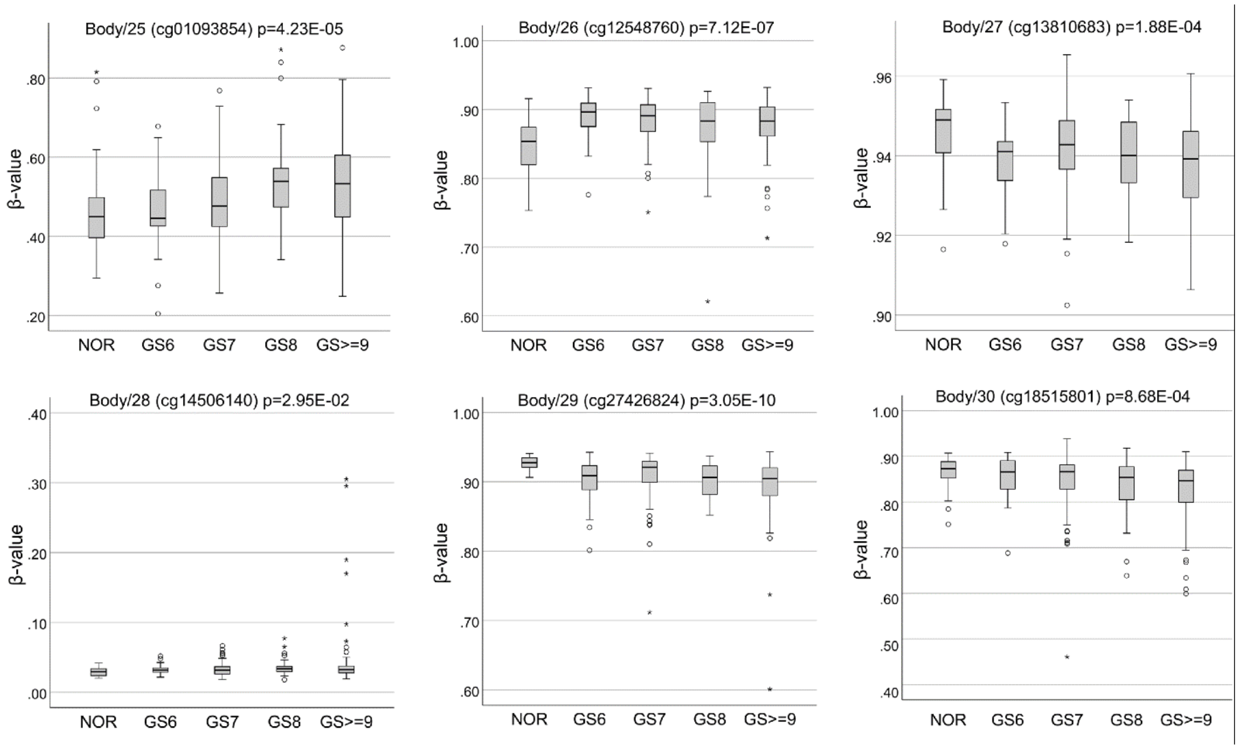
Body/25 (cg01093854) p=4.23E-05
Body/26 (cg12548760) p=7.12E-07
Body/27 (cg13810683) p=1.88E-04
Body/28 (cg14506140) p=2.95E-02
Body/29 (cg27426824) p=3.05E-10
Body/30 (cg18515801) p=8.68E-04
β-value
NOR
GS6
GS7
GS8
GS>=9

Supplementary Figure 4) Determination of epigenetic reasons for *TET1*-overexpression in PCa. Methylation values (ß-values) in 30 CpG-probes within the promoter and gene body of *TET1* gene were analyzed using TCGA database in PCa samples with high *TET1* expression (*TET1*-HIGH, expression level above 85th percentile, n=51) and PCa with low *TET1* expression (*TET1*-LOW, expression level under 40th percentile, n=136), and correlation analyses between *TET1*-CpGs' methylation and *TET1* expression were done. **A)** *TET1*-HIGH PCa showed at four CpG-sites a significant hypomethylation in comparison to *TET1*-LOW PCa (Mann-Whitney-U test), and hypomethylation at these four CpG sites was significantly negative correlated to *TET1* expression, i.e. contributed to an increased *TET1* expression (Spearman correlation, s. STable 2); **B)** *TET1*-HIGH PCa showed at three CpG-sites a significant hypermethylation in comparison to *TET1*-LOW PCa (Mann-Whitney-U test), and hypermethylation at these three CpG sites was significantly positive correlated to *TET1* expression, i.e. also contributed to an increased *TET1* expression (Spearman correlation, s. STable 2).

A

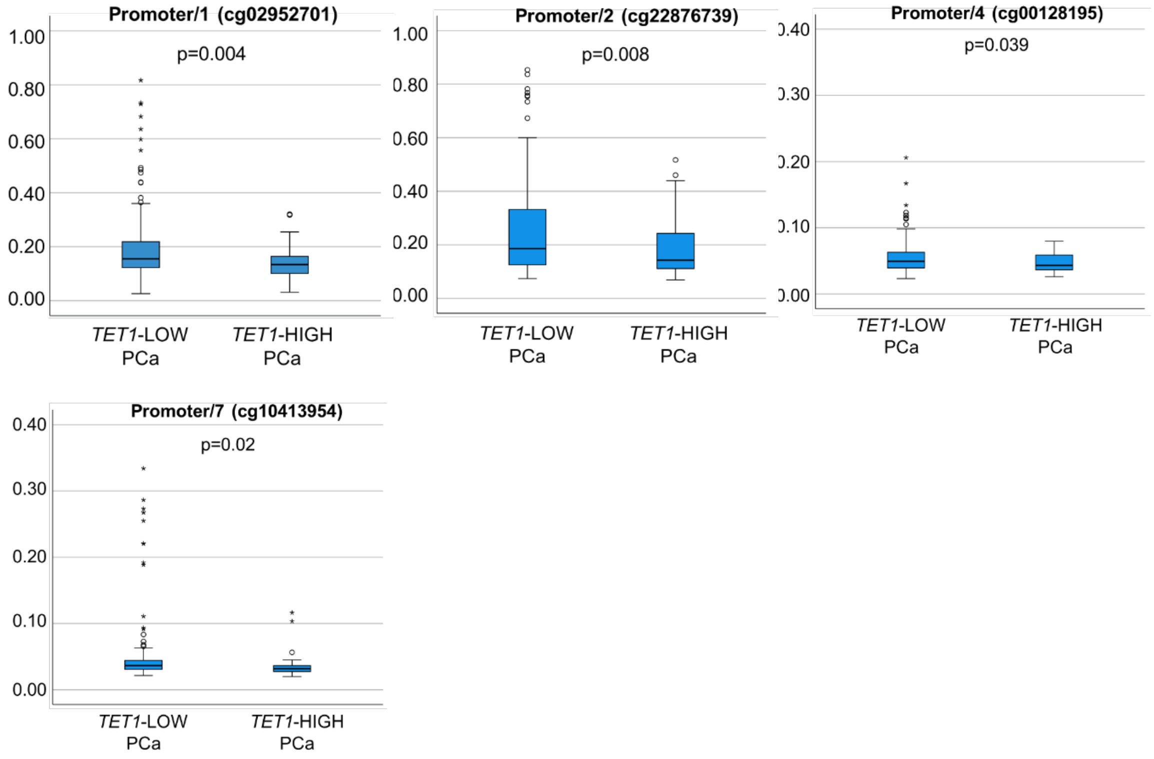

B

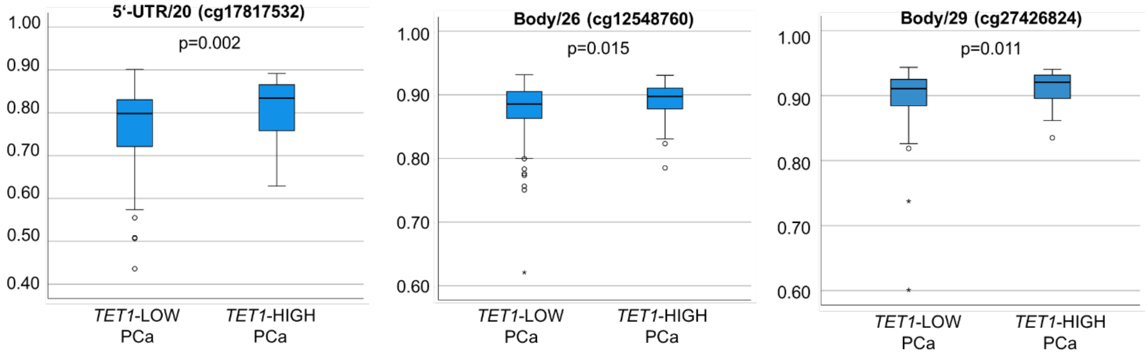

Supplementary Figure 5) Analysis of DNA binding motifs of *TET1*-correlated transcription factors (TFs). Binding motifs of TFs overexpressed in PCa in a significant positive correlation to *TET1* expression were analyzed using JASPAR2020 database. Binding motifs of 21 out of 161 *TET1*-correlated TFs were available in the database (CpG-sensitive TFs, i.e. TFs exhibiting CpG-sites in their binding motif, are framed in red, n=9).

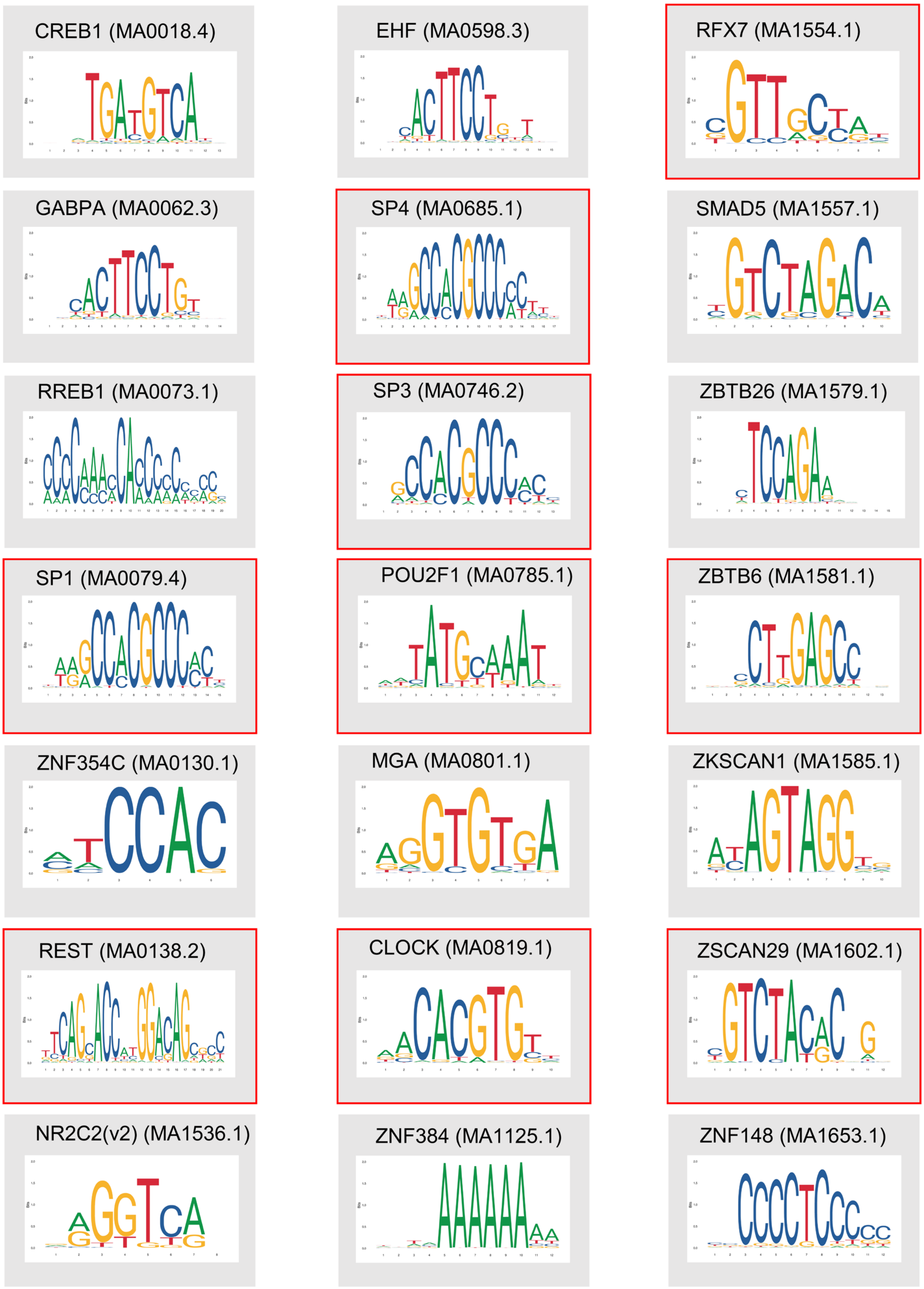

Supplementary Figure 6) Protein-protein interaction (interactome) analyses of 161 *TET1*-coactivated TFs in PCa with a focus on SP1 and CREB1. A) SP1 showed significant binding sites in *TET1*-promoter (promoter/7, significantly hypomethylated in *TET1*-HIGH PCa and responsible for *TET1*-overexpression) and in promoters of 624 out of a total of 626 *TET1*-coactivated genes in PCa. Among 161 *TET1*-coactivated TFs, six were found to directly interact with SP1 (**A.1**), and 33 were found to interact with SP1 through a bridge TF (**A.2**); **B)** CREB1 showed significant binding sites in *TET1*-promoter (promoter/1 and promoter/2, significantly hypomethylated in *TET1*-HIGH PCa and also responsible for *TET1*-overexpression) and in promoters of 533 out of a total of 626 *TET1*-coactivated genes. Among 161 *TET1*-coactivated TFs, three were found to directly interact with CREB1 (**B.1**), and 31 were found to interact with CREB1 through a bridge TF (**B.2**) (respective families of TFs are shown in the legend in different colors).

A.1

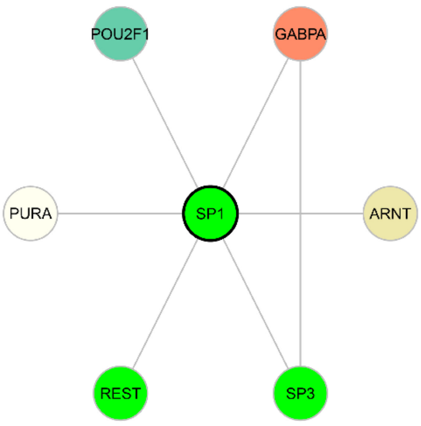

A.2

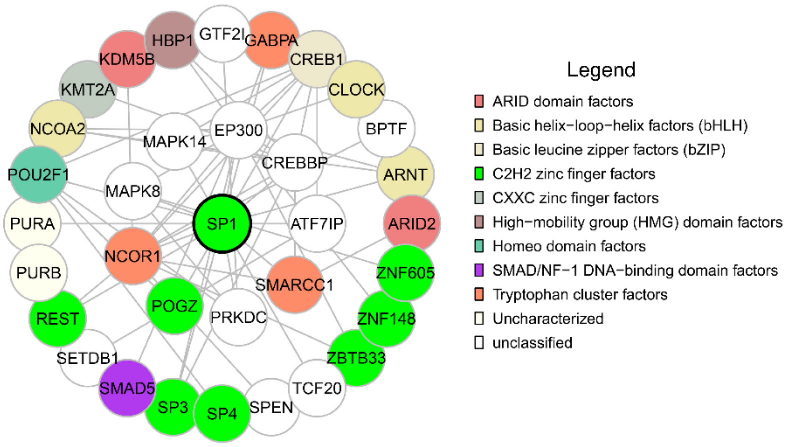

B.1

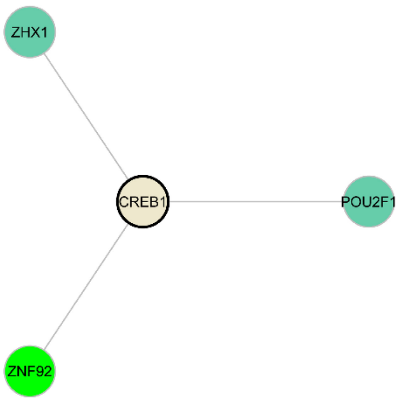

B.2

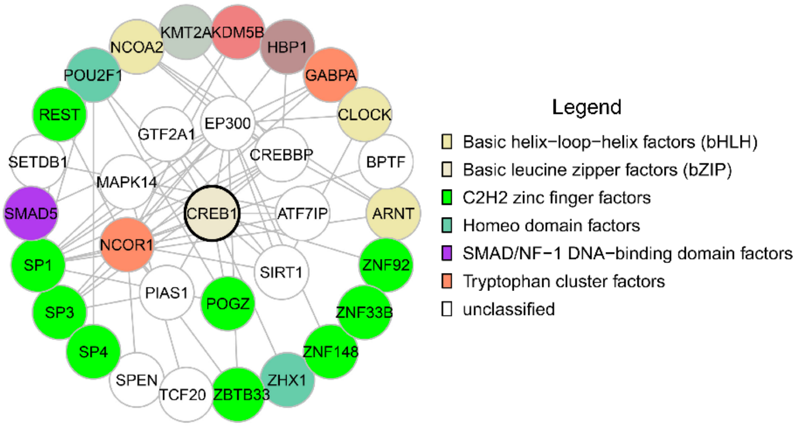

Supplementary Figure 7) Mutual correlation of expression and promoter methylation of *TET1* and *TET1*-coactivated genes in PCa. Genes enriched under the gene ontology terms “Covalent chromatin modification”, “Histone modification” and “Peptidyl-lysine-modification” (Epigenetic Modifier) and hallmarks “Mitotic spindle”, “G2M checkpoint” and “E2F targets” (Mitotic Regulators) are shown. Expression of all genes showed a strong positive mutual correlation (upper half of the diagram: blue color indicates a significant positive correlation, Pearson correlation). *TET1*-expression was significantly negative correlated to the promoter methylation of the coactivated genes, and expression of all genes was significantly negative correlated to own promoter methylation (black squares in the lower half of the diagram: orange color indicates a significant negative correlation, Spearman correlation).

Genes	Function	*Genes*	Function
ATM	Epigenetic Modifier	*NUP205*	Both functions
ATF7IP	Epigenetic Modifier	*WRN*	Mitotic Regulator
KDM3A	Epigenetic Modifier	*ASH1L*	Epigenetic Modifier
RASA1	Mitotic Regulator	*EPC2*	Epigenetic Modifier
HMG20A	Epigenetic Modifier	*IPO7*	Mitotic Regulator
REST	Epigenetic Modifier	*UBR5*	Epigenetic Modifier
RICTOR	Mitotic Regulator	*KDM1B*	Epigenetic Modifier
KAT6B	Epigenetic Modifier	*CCP110*	Mitotic Regulator
ATRX	Both functions	*NUP153*	Both functions
PIAS1	Epigenetic Modifier	*RIF1*	Epigenetic Modifier
SETD2	Epigenetic Modifier	*SMC3*	Both functions
NIPBL	Epigenetic Modifier	*YTHDC1*	Mitotic Regulator
ARHGAP5	Mitotic Regulator	*NUP160*	Epigenetic Modifier
LATS1	Mitotic Regulator	*EPC1*	Epigenetic Modifier
CASP8AP2	Mitotic Regulator	*SIRT1*	Epigenetic Modifier
BCOR	Epigenetic Modifier	*KMT2E*	Epigenetic Modifier
BAZ2A	Epigenetic Modifier	*DNMT3A*	Epigenetic Modifier
HUWE1	Epigenetic Modifier	*TASOR*	Epigenetic Modifier
ALS2	Mitotic Regulator	*KDM7A*	Epigenetic Modifier

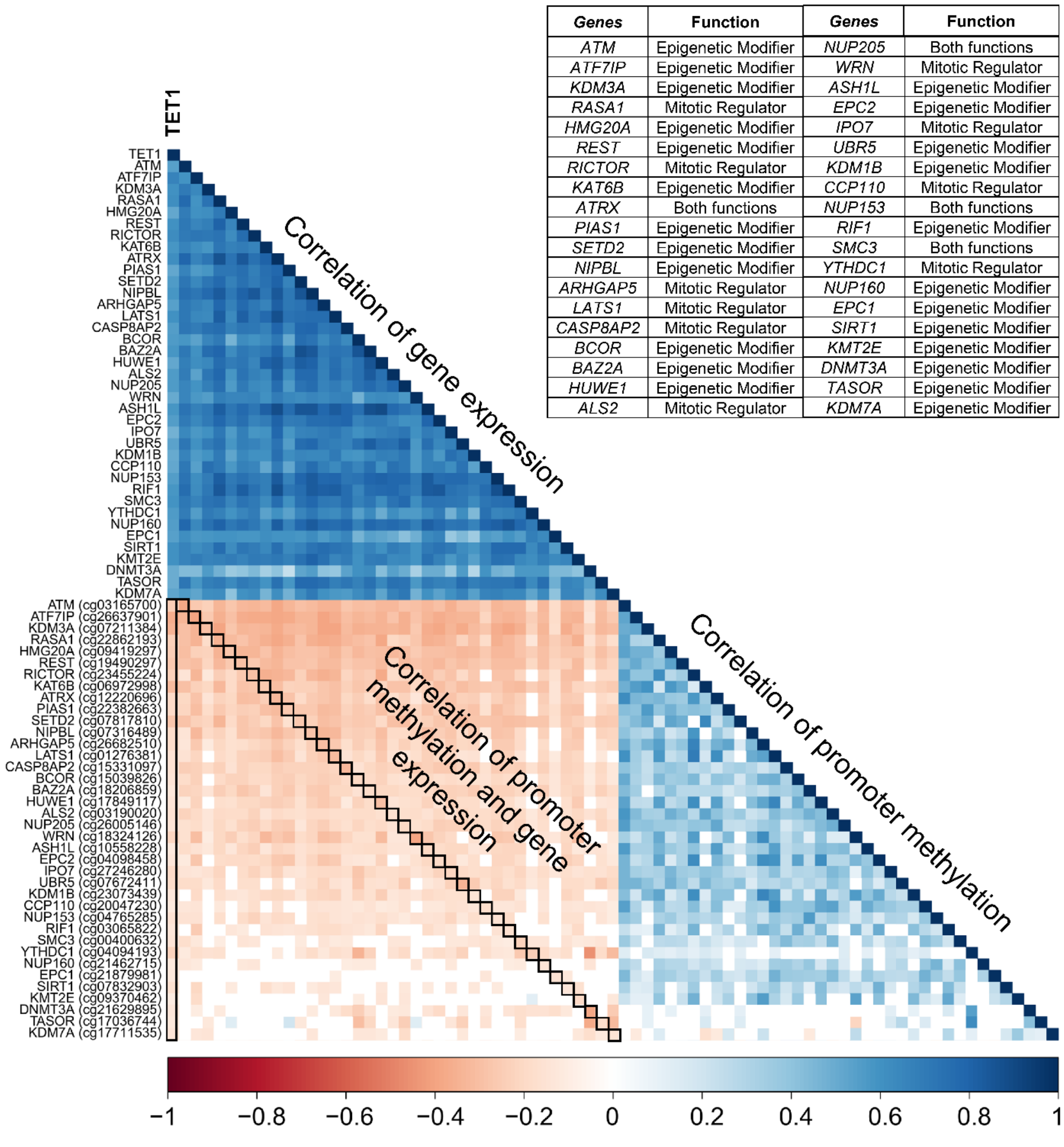

Supplementary Figure 8) Strong positive correlation between gene expression of DNA demethylases of TET family and DNA methyltransferases in normal prostate and PCa. A) In normal prostate, significantly positive correlation was found between expression of *TET1*, *TET2* and *TET3,* and between *TET1* and DNA methyltransferase 1 gene (*DNMT1*); **B)** In PCa, *TET1* expression was significantly positive correlated to expression of *TET2*, *TET3* and *DNMT1* as well as to expression of *DNMT3A* and *DNMT3B* (Pearson correlation, correlation coefficients and 2-tailed p-values are given).

A

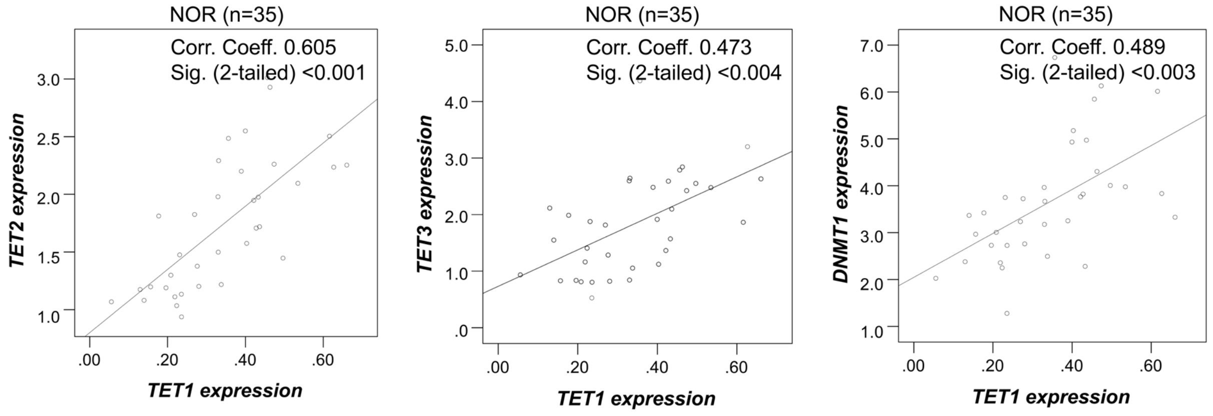

B

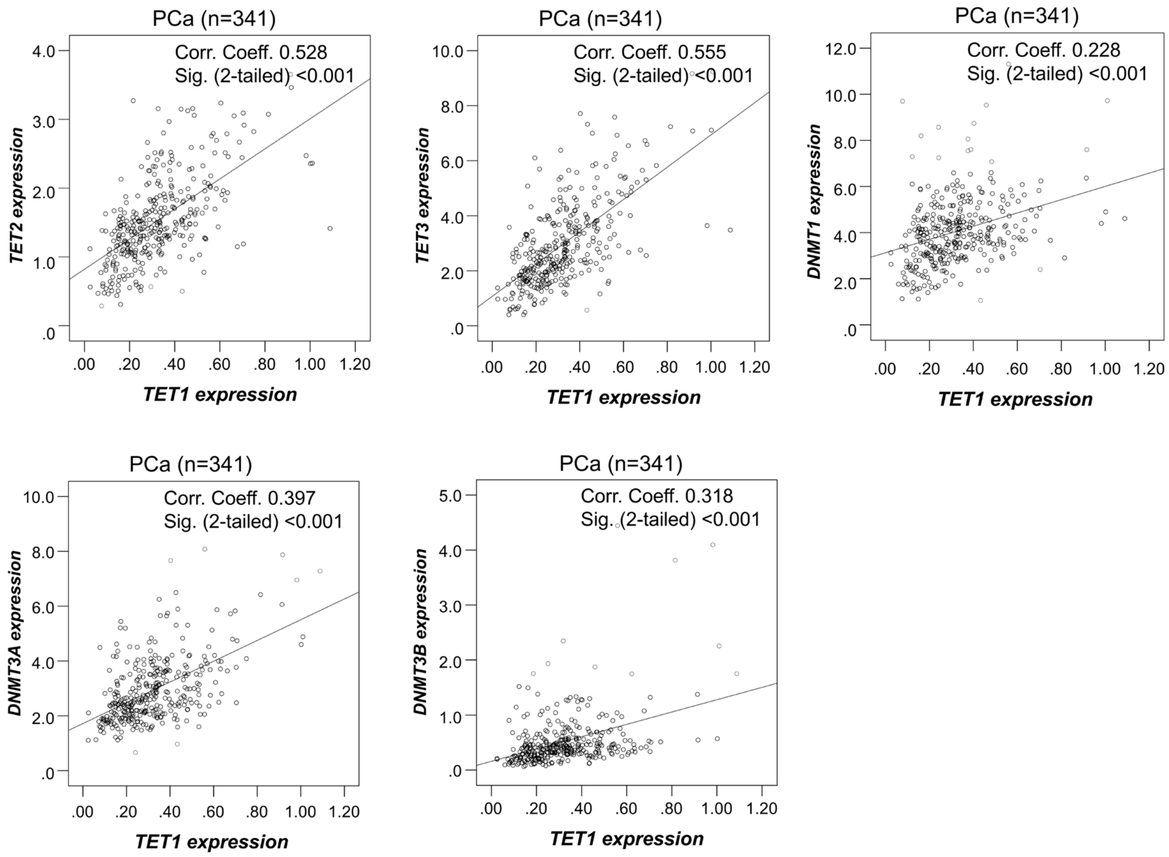

Supplementary Figure 9) Gene set enrichment analysis of “Oncogenic signature gene sets” on 626 *TET1*-coactivated genes. This Molecular signature Database (MSigDB) gene set represent signatures of cellular pathways, which are often dysregulated in cancer [35]. Highly significant enrichments were found for TBK1.DF DN (genes down-regulated in epithelial lung cancer cell lines upon overexpression of an oncogenic form of KRAS (Kirsten rat sarcoma viral oncogene homolog) gene and knockdown of TBK1 (TANK Binding Kinase 1) gene by RNA-interference, RNAi), PGF UP.V1_UP (genes up-regulated in human umbilical vein endothelial cells, HUVEC, by treatment with Placental Growth Factor, PGF), JAK2 DN.V1 DN (genes down-regulated in human erythroleukemia cells after knockdown of Janus Kinase 2, JAK2, gene by RNAi), BCAT BILD ET AL DN (genes down-regulated in primary epithelial breast cancer cell culture overexpressing activated Catenin Beta 1, CTNNB1, gene) and VEGF A UP.V1 DN (genes down-regulated in HUVEC cells by treatment with Vascular Endothelial Growth Factor A, VEGFA) [35].

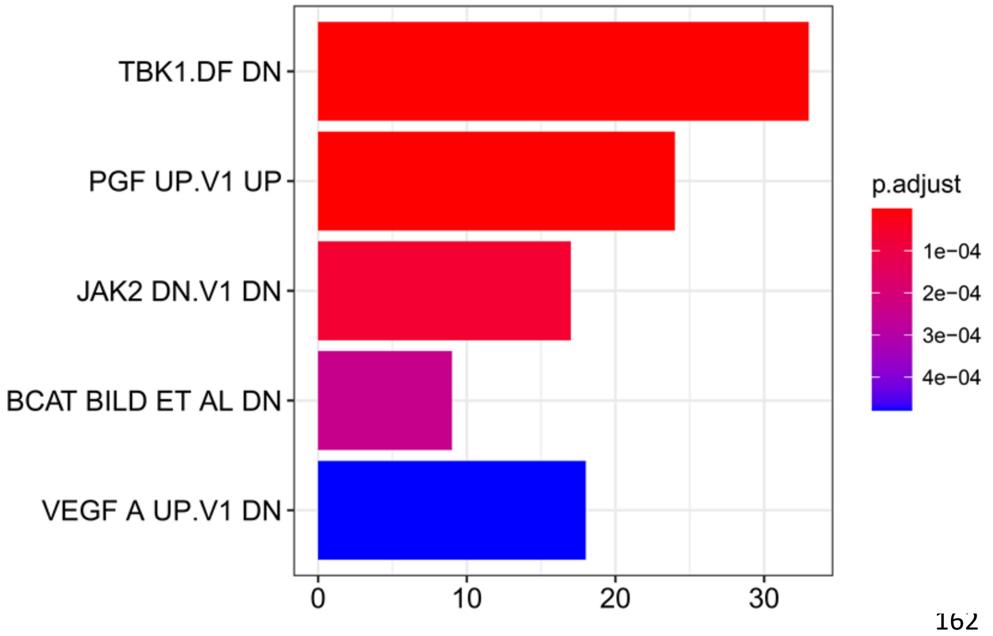

Supplementary Figure 10) Kyoto Encyclopedia of Genes and Genomes (KEGG) pathway analysis of *TET1*-coactivated genes in PCa. Significantly positive *TET1*-correlated and coactivated genes in PCa (r>0.5, Pearson correlation, n=626 genes in total) were analyzed for KEGG pathway enrichment. **A)** Significantly enriched KEGG pathways are shown together with numbers of involved genes (lower row) and adjusted p-values; **B)** Significantly enriched KEGG pathways are shown together with specific genes; **C and D)** The most significantly enriched KEGG pathways potentially involved in carcinogenesis and basal cell function, "Herpes simplex virus 1 infection" and "Signaling pathways regulating pluripotency of stem cells" are illustrated with main processes and components, respectively, and involved genes (*ZAPs*: genes encoding zinc-finger antiviral proteins).

A

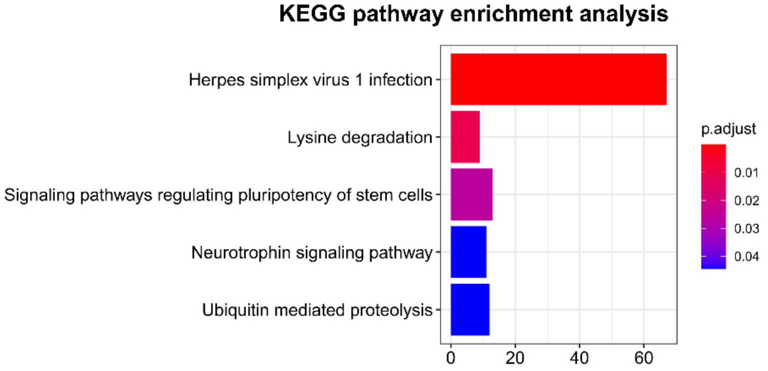

B

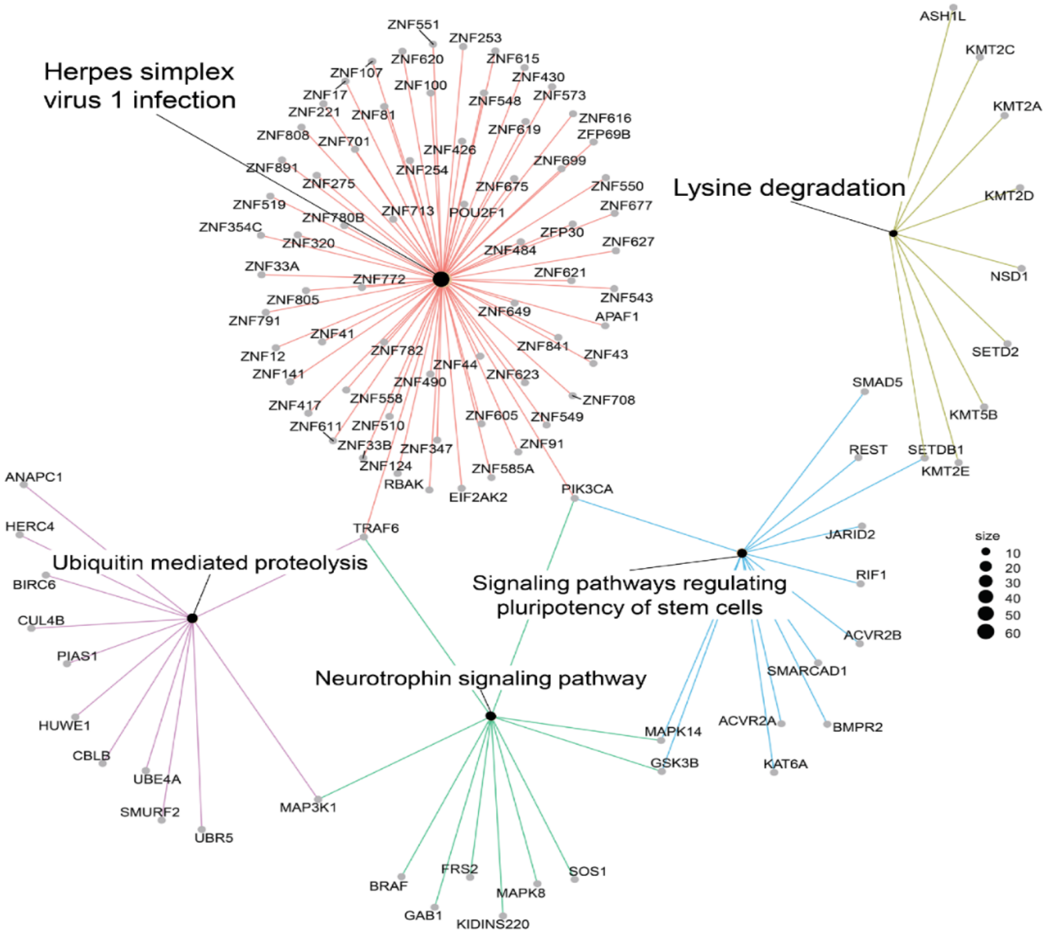

C

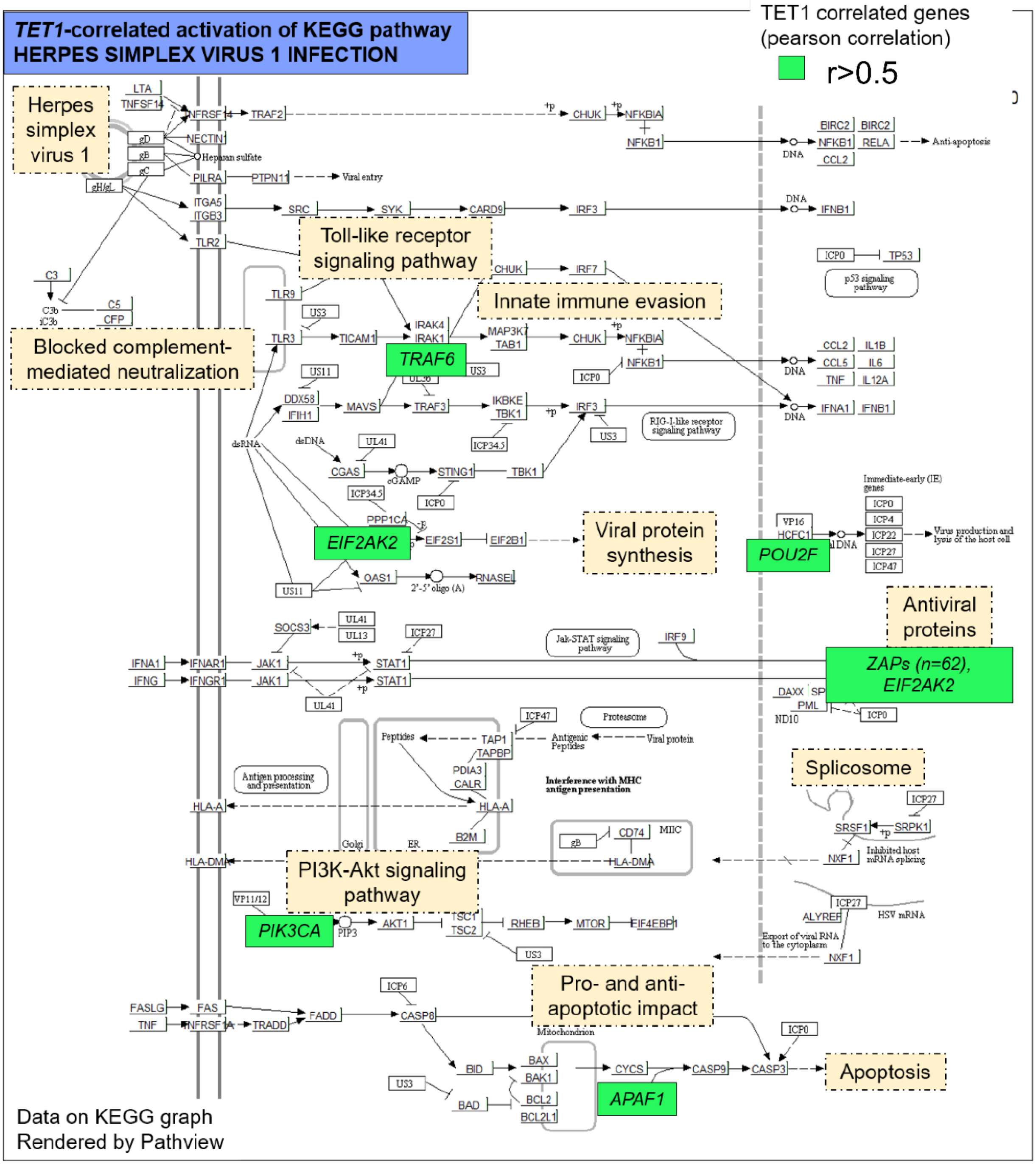

TET1-correlated activation of KEGG pathway
HERPES SIMPLEX VIRUS 1 INFECTION
TET1 correlated genes
(pearson correlation)
r>0.5
Herpes simplex virus 1
Toll-like receptor signaling pathway
Innate immune evasion
Blocked complement-mediated neutralization
TRAF6
EIF2AK2
Viral protein synthesis
POU2F
Antiviral proteins
ZAPs (n=62), EIF2AK2
Splicosome
PI3K-Akt signaling pathway
PIK3CA
Pro- and anti-apoptotic impact
APAF1
Apoptosis
Data on KEGG graph
Rendered by Pathview

D

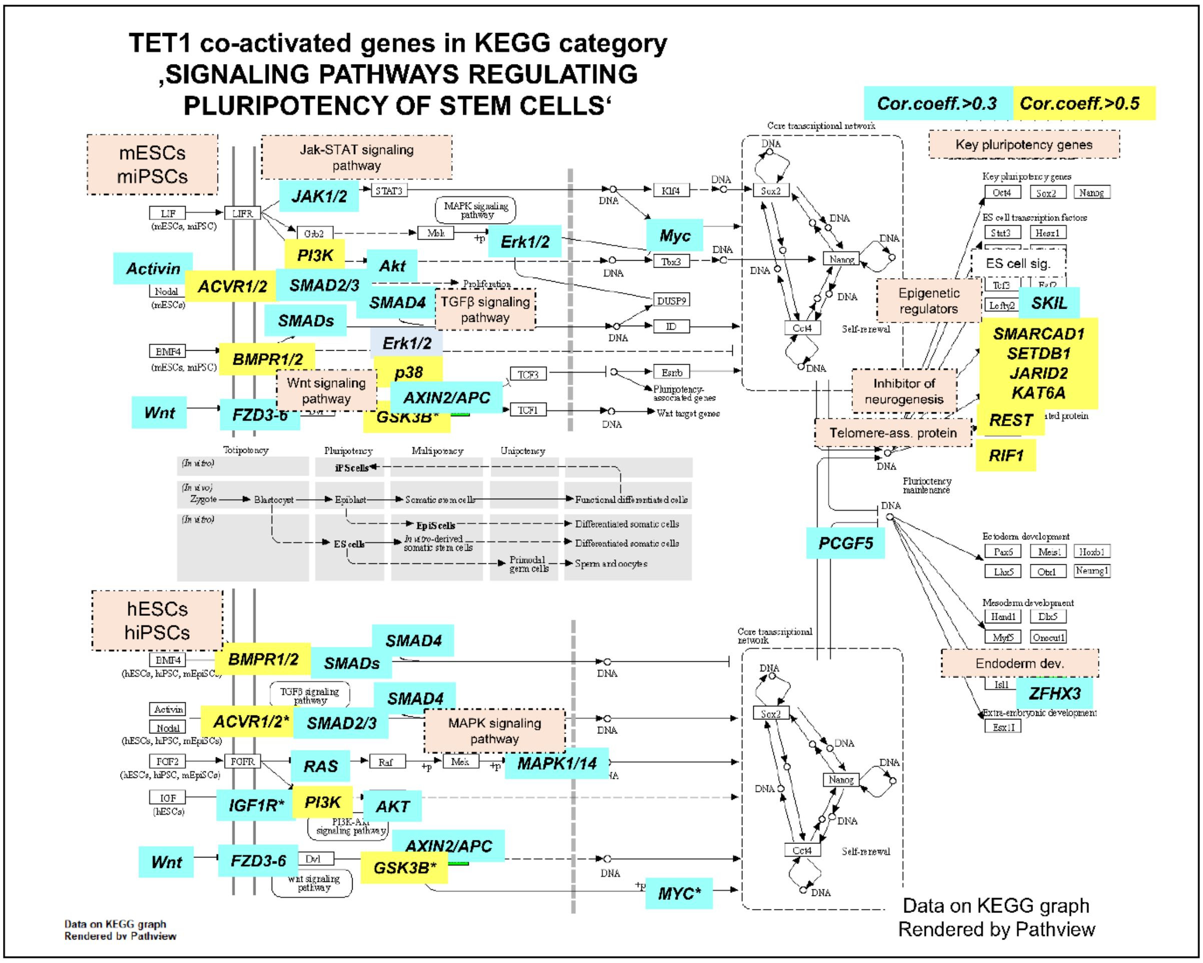
TET1 co-activated genes in KEGG category ‚SIGNALING PATHWAYS REGULATING PLURIPOTENCY OF STEM CELLS‘
Cor.coeff.>0.3
Cor.coeff.>0.5
mESCs miPSCs
Jak-STAT signaling pathway
JAK1/2
Activin
ACVR1/2
PI3K
Akt
SMAD2/3
SMAD4
TGFβ signaling pathway
SMADs
Erk1/2
BMPR1/2
p38
Wnt signaling pathway
AXIN2/APC
Wnt
FZD3-6
GSK3B*
Erk1/2
Myc
Key pluripotency genes
Epigenetic regulators
ES cell sig.
SKIL
SMARCAD1
SETDB1
JARID2
KAT6A
Inhibitor of neurogenesis
REST
Telomere-ass. protein
RIF1
PCGF5
Endoderm dev.
ZFHX3
hESCs hiPSCs
BMPR1/2
SMADs
SMAD4
ACVR1/2*
SMAD2/3
MAPK signaling pathway
RAS
MAPK1/14
IGF1R*
PI3K
AKT
AXIN2/APC
Wnt
FZD3-6
GSK3B*
MYC*
Data on KEGG graph
Rendered by Pathview
