# Supplementary material for: Tracing TET1 expression in prostate cancer: discovery of malignant cells with a distinct oncogenic signature
Source: Clin Epigenetics. 2021 Nov 29;13:211. doi: 10.1186/s13148-021-01201-7 (PMC8630881; doi:10.1186/s13148-021-01201-7)
Supplement: Supplementary file 2 — Additional file 2. Supplementary Tables. [file 13148_2021_1201_MOESM2_ESM.pdf]

Supplementary Table 1 Clinical data of PCa patients in TCGA database used for gene regulation network and functional genomics studies (n=341 patients in total)

	Count (%)	Age (y)		PSA (ng/ml)	
		**median**	**range**	**median**	**range**
**T2a-c**	130 (38.1%)	60.4	42.0-77.9	6.1	2.2-18.1
**T3a-b**	199 (58.4%)	62.7	44.5-78.6	8.5	1.6-70
**T4**	5 (1.5%)	68.3	64.3-71.8	17.1	4.6-87
**NA**	7 (2.0%)	64.6	51.1-73.9	10.3	10-10.6
**GS6**	36 (10.6%)	58.3	42.0-77.9	6.3	2.2-12.8
**GS7**	158 (46.3%)	61.3	43.5-75.1	6.5	1.6-87
**GS8**	39 (11.4%)	64.8	50.4-74.0	9.0	4.7-51.7
**GS>=9**	108 (31.7%)	64.4	44.5-78.6	11.5	2.2-70

Abbreviations: PCa: prostate cancer; TCGA: The Cancer Genome Atlas; y: years; PSA: prostate-specific antigen; T: tumor stage; GS: Gleason score.

Supplementary Table 2 Thirty CpG-probes available for methylation analyses in *TET1* gene promoter (TSS1500 and TSS200), 5’-UTR and gene body using Illumina 450k microarray

***TET1*-CpG-probe / ID**	***TET1*-CpG-locus / Nr.**	**Sequence**	**Strand**
cg02952701	Promoter/1	TGAAGAAGGTGCCAGGTCAGAGATGACACCATTGAGCAATGTCTAGGTCG	neg.
cg22876739	Promoter/2	TGCCAGGTCAGAGATGACACCATTGAGCAATGTCTAGGTCGCCAGCAACG	neg.
cg23602092	Promoter/3	AAACTTAGCTCTTCCTGCCCTTTCATTTCTTCATTCATAAAGTGAATACG	pos.
cg00128195	Promoter/4	CGAGGAGGGATTCCAGCTCCAGTTTGGGTAAATCCAGCTCGCGTTTTGTC	pos.
cg05400741	Promoter/5	GTGTCTCCCCAGGACAGACCTCAGGGAGTGAAGCTGGGGCTCTGCCTTCG	neg.
cg07669489	Promoter/6	CGCGTTTTGTCTCTCGCTCAACTGTGCAGGGTCCAGCGAAGGCAGAGCCC	pos.
cg10413954	Promoter/7	TGGGGCTCTGCCTTCGCTGGACCCTGCACAGTTGAGCGAGAGACAAAACG	neg.
cg21948169	Promoter/8	AGGGATTCCAGCTCCAGTTTGGGTAAATCCAGCTCGCGTTTTGTCTCTCG	pos.
cg03651138	5'-UTR/9	CGGGTCTGCCCCCCGGGACACCCCTCTGCCTCGCCCAAGTCATGCAGCCC	pos.
cg19439331	5'-UTR/10	CGCACGGCCCCCAGCTCCAAGCCTGCACCAGCCCTCGGGCAAACTTTCCA	neg.
cg02774862	5'-UTR/11	CGAGAAACCCACGCTGGGCATTTCTGATCCACTAAAAACTAAAGTGTTTC	pos.
cg03756448	5'-UTR/12	TGTGACCCGGCCAGCGGTGAGTTGGGGCCGGGGCAGAGGGCAGGGGTGCG	pos.
cg06767766	5'-UTR/13	AGGAGAGCATTTTTTCTCTGGGGGCGGTAGGTTGTGCGGCCGCCTCCTCG	neg.
cg09183181	5'-UTR/14	GGGACGCTTTTGTTGCCGGAGATAAACAATGCCCCCGGCCGGAGGTATCG	pos.
cg09978996	5'-UTR/15	AAAGTGTTTCCAAAAGAACACTTAAAGCTTCAGCCTCGGAGTCTTTCCCG	pos.
cg10403849	5'-UTR/16	CGCCGAGCACACTCAGGTCCAGGGGAGACCTGGGGCTGCTGCTATACAGC	neg.
cg12630147	5'-UTR/17	CTTTAGTTTTTAGTGGATCAGAAATGCCCAGCGTGGGTTTCTCGTTCACG	neg.
cg13848707	5'-UTR/18	TTCTGTCAAAATGGCATTTAAGAAACTCATTTATGAGCACCACTGGGTCG	pos.
cg15254238	5'-UTR/19	GCTGCAAGACCTTGGAGCGAGAACAGGATTAGGTACACAGCCCTGGGCCG	pos.
cg17817532	5'-UTR/20	TGTTATGTCTTTCTTGTCTTCGGAAGGACAGTTCTATGTCCATTCTAACG	pos.
cg19127638	5'-UTR/21	CTTACAACTCTGGGACTGCCACTTTCCATTTTAATAAGAGTTAACTATCG	pos.
cg20326704	5'-UTR/22	CGAGCTGCACAATATGTCCTAGTAATGACTGCTTTTTGAAGAAATGTTTG	pos.
cg23350336	5'-UTR/23	GAAATGCCCAGCGTGGGTTTCTCGTTCACGTGGAAAGATTACCAGCGTCG	neg.
cg25926515	5'-UTR/24	AGCGAGAACAGGATTAGGTACACAGCCCTGGGCCGGCGGGGTGAGCCGCG	pos.
cg01093854	Body/25	CGGTAGTGGACTCTGGCTTAAAAGAAAAAGAATAAAAGTGCCTGTGGGTG	pos.
cg12548760	Body/26	AATTCATGCGTGCTGCTCCAGCTCTTGTCAGAAGGCTTCTGACTGGCACG	neg.
cg13810683	Body/27	CGCAACAGCCTCATGCGGGTTTTCAGAAAGAAGCAGCACTCCCCACTGTA	pos.
cg14506140	Body/28	CGGCTCGGCTCTCACCACAGGACACAGGCAGCCACAAGCTGCGACATGGA	neg.
cg27426824	Body/29	CGGCCATTGGAAGAGGGATGCCATCCCACACCATGATGAGCACCACCATC	neg.
cg18515801	Body/30	CGGGGTGGGTATTCTTAACTGTGACTATATTTTGACAATTGGTAGAAGGT	pos.

Abbreviations: TSS: transcription start site; 5‘-UTR: 5‘-untranslated region; ID: identification in Illumina Human Methylation 450k microarray; Nr.: number of CpG-probe; neg.: negative; pos.: positive

Supplementary Table 3 Comparison of methylation values (ß-values) in 30 *TET1* CpG-probes between normal prostate (NOR, n=35) and prostate carcinoma (PCa, n=341), and correlation analyses between *TET1*-methylation and -expression in NOR and PCa

TET1-CpG-locus / Nr.	Median ß-value NOR	Median ß-value PCa	Differential methylation in PCa	Diff. methyl. adjusted p-value	Corr. p-value M-E / NOR	Corr. coeff. M-E / NOR	Corr. p-value M-E / PCa	Corr. coeff. M-E / PCa
**Promoter/1**	**0.105**	**0.141**	**hypermeth**	**1.57E-04**	**0.009**	**-0.433**	**<0.0001**	**-0.223**
**Promoter/2**	**0.124**	**0.154**	**hypermeth**	**1.65E-03**	0.153	-0.246	**<0.001**	**-0.203**
**Promoter/3**	**0.436**	**0.514**	**hypermeth**	**1.16E-03**	0.478	-0.124	0.503	-0.036
Promoter/4	0.051	0.049	no change	8.28E-01	0.623	0.086	**0.031**	**-0.117**
Promoter/5	0.041	0.040	no change	5.94E-01	0.634	-0.083	0.195	-0.070
**Promoter/6**	**0.018**	**0.021**	**hypermeth**	**2.46E-02**	0.737	-0.059	0.826	-0.012
Promoter/7	0.031	0.035	no change	1.45E-01	0.061	-0.320	**0.001**	**-0.172**
Promoter/8	0.050	0.042	no change	8.41E-01	0.226	-0.210	0.923	-0.005
5'-UTR/9	0.045	0.044	no change	5.43E-01	0.422	0.140	0.181	-0.073
5'-UTR/10	0.040	0.042	no change	7.40E-01	**0.038**	**-0.353**	0.527	-0.034
**5'-UTR/11**	**0.139**	**0.557**	**hypermeth**	**3.69E-23**	0.654	-0.078	0.507	0.036
5'-UTR/12	0.025	0.028	no change	1.82E-01	0.125	-0.264	0.978	-0.002
**5'-UTR/13**	**0.062**	**0.394**	**hypermeth**	**1.15E-21**	**0.016**	**-0.405**	0.514	-0.035
**5'-UTR/14**	**0.109**	**0.096**	**hypometh**	**4.42E-02**	0.881	0.026	**0.042**	**-0.110**
**5'-UTR/15**	**0.329**	**0.559**	**hypermeth**	**1.11E-13**	0.861	0.031	0.067	0.099
**5'-UTR/16**	**0.618**	**0.738**	**hypermeth**	**3.33E-07**	0.431	-0.138	0.099	0.089
**5'-UTR/17**	**0.112**	**0.628**	**hypermeth**	**2.55E-27**	0.636	-0.083	0.177	0.073
**5'-UTR/18**	**0.045**	**0.264**	**hypermeth**	**6.82E-11**	0.209	-0.218	**0.046**	**-0.108**
**5'-UTR/19**	**0.426**	**0.650**	**hypermeth**	**1.22E-10**	0.837	-0.036	0.132	0.082
**5'-UTR/20**	**0.600**	**0.805**	**hypermeth**	**9.62E-19**	**0.047**	**-0.338**	**0.006**	**+0.147**
**5'-UTR/21**	**0.812**	**0.871**	**hypermeth**	**4.74E-10**	0.055	-0.327	0.087	0.093
**5'-UTR/22**	**0.172**	**0.577**	**hypermeth**	**3.49E-17**	0.691	-0.070	0.172	0.074
**5'-UTR/23**	**0.080**	**0.523**	**hypermeth**	**3.40E-21**	0.898	-0.022	0.347	0.051
**5'-UTR/24**	**0.489**	**0.610**	**hypermeth**	**3.38E-06**	0.388	-0.151	0.231	0.065
Body/25	0.450	0.503	no change	1.56E-01	0.150	-0.249	0.252	-0.062
**Body/26**	**0.853**	**0.888**	**hypermeth**	**1.37E-07**	0.237	-0.205	**0.003**	**+0.160**
**Body/27**	**0.949**	**0.941**	**hypometh**	**1.72E-03**	0.201	-0.221	0.618	0.027
**Body/28**	**0.029**	**0.032**	**hypermeth**	**3.09E-02**	0.440	-0.135	0.068	-0.099
**Body/29**	**0.927**	**0.912**	**hypometh**	**2.98E-05**	0.243	0.203	**0.022**	**+0.124**
**Body/30**	**0.873**	**0.861**	**hypometh**	**3.35E-02**	0.352	-0.162	**0.043**	**+0.109**

Abbreviations and codes: 5‘-UTR: 5‘-untranslated region; Adjusted p-value; Diff. methyl.: differential methylation (adjusted p-values were calculated using eBayes function of R); Corr.: Spearman correlation; M-E: correlation between methylation (M) and expression (E); Corr. coeff.: correlation coefficient (negative “-“ and positive “+” correlations are indicated); hypermeth (red): 25 CpG-loci significantly hypermethylated in PCa; hypometh (green): 5 CpG-loci significantly hypomethylated in PCa; yellow background: significant correlations found between *TET1*-CpG-methylation and *TET1*-expression in NOR and/or PCa

Supplementary Table 4 *TET1*-correlated and -coactivated TFs in PCa were analyzed with regard to the binding sites in *TET1* gene using JASPAR2020 (using available data, 21 TFs exhibiting a significant, i.e. true, binding site were identified)

***TET1*-CpG-locus / Nr.**	**ß-value *TET1*-LOW PCa**	**ß-value *TET1*-HIGH PCa**	**Diff. meth. *TET1*-LOW vs. -HIGH PCa**	**Effect on *TET1* expr.**	**TFs and TF-binding sites in *TET1* gene (JASPAR2020, p<0.01*)**
**Promoter/1**	**0.16**	**0.13**	**0.004**	**Upreg.**	**CREB1, MGA, NR2C2(var.2), SMAD5, ZBTB6**
**Promoter/2**	**0.19**	**0.14**	**0.008**	**Upreg.**	**CREB1, MGA, NR2C2(var.2), RFX7, SMAD5, ZBTB6, ZSCAN29**
Promoter/3	0.54	0.54	0.873		GABPA, ZNF354C, EHF, NR2C2(var.2)
**Promoter/4**	**0.05**	**0.04**	**0.039**	**Upreg.**	**ZNF354C, CLOCK, NR2C2(var.2), ZBTB6**
Promoter/5	0.04	0.04	0.117		GABPA, SP1, ZNF354C, SP3, MGA, SMAD5, ZSCAN29
Promoter/6	0.02	0.02	0.744		ZNF354C, MGA, NR2C2(var.2), ZBTB6, ZSCAN29
**Promoter/7**	**0.04**	**0.03**	**0.002**	**Upreg.**	**SP1, ZNF354C, MGA, NR2C2(var.2), ZBTB6, ZSCAN29**
Promoter/8	0.04	0.04	0.791		ZNF354C, CLOCK, NR2C2(var.2), ZBTB6
5-UTR/9	0.04	0.04	0.391		SP1, SP3, NR2C2(var.2), SMAD5, ZNF148
5-UTR/10	0.04	0.04	0.292		ZNF354C, NR2C2(var.2), RFX7
5-UTR/11	0.56	0.62	0.232		SP1, ZNF354C, MGA, ZNF384, RFX7, ZSCAN29
5-UTR/12	0.03	0.03	0.189		SP1, SP4, SP3, NR2C2(var.2), ZNF148
5-UTR/13	0.40	0.39	0.651		ZNF354C, SP3, CLOCK, ZNF384, NR2C2(var.2), ZNF148
5-UTR/14	0.10	0.10	0.196		SP1, SP3, NR2C2(var.2), RFX7
**5-UTR/15**	**0.55**	**0.59**	**0.030**	No effect	MGA, ZNF384, RFX7, ZBTB26, ZBTB6
5-UTR/16	0.73	0.76	0.103		ZNF354C, MGA, RFX7
5-UTR/17	0.59	0.71	0.053		SP1, ZNF354C, ZNF384, NR2C2(var.2), RFX7, ZSCAN29
5_UTR/18	0.30	0.19	0.128		ZNF354C, CLOCK, ZNF384
5-UTR/19	0.63	0.70	0.057		ZNF354C, MGA,
**5-UTR/20**	**0.80**	**0.83**	**0.002**	**Upreg.**	**ZNF384, ZSCAN29**
**5-UTR/21**	**0.86**	**0.88**	**0.043**	No effect	POU2F1
5-UTR/22	0.57	0.65	0.080		ZNF354C, CLOCK, ZNF384,
5-UTR/23	0.49	0.61	0.138		ZNF354C, CLOCK, NR2C2(var.2), RFX7, ZSCAN29
5-UTR/24	0.60	0.64	0.176		SP3, MGA, NR2C2(var.2), ZNF148
Body/25	0.52	0.50	0.328		ZNF354C, CLOCK, ZNF384
**Body/26**	**0.89**	**0.90**	**0.015**	**Upreg.**	**ZNF354C, CLOCK, RFX7, ZBTB6**
Body/27	0.94	0.94	0.740		SP1, ZNF354C, SP3, RFX7
Body/28	0.03	0.03	0.367		ZNF354C, MGA, CLOCK, NR2C2(var.2), RFX7
**Body/29**	**0.91**	**0.92**	**0.011**	**Upreg.**	**ZNF354C, MGA**
Body/30	0.85	0.86	0.113		ZNF354C, MGA, ZNF384, NR2C2(var.2)

Abbreviations and codes: TF: transcription factor; 5‘-UTR: 5‘-untranslated region; Upreg.: results in upregulation; Differential methylation of *TET1*-LOW versus -HIGH expressing PCa

was analyzed by Mann-Whitney-U test (asymptotic significance 2-sided is given); *p-value: significance of TF-binding was calculated according to Touzet and Varre (Touzet and Varre, 2007) and $p<0.01$ was considered as statistically significant; green: CpG-loci significantly hypomethylated in *TET1*-HIGH PCa and showing a significant negative correlation to *TET1*-expression, and causing an upregulation of *TET1*-expression; red: CpG-loci significantly hypermethylated in *TET1*-HIGH PCa and showing a significant positive correlation to *TET1*-expression, and causing an upregulation of *TET1*-expression

Supplementary Table 5 Binding characteristics of *TET1*-coactivated TFs in *TET1*-promoter and promoters of *TET1*-coactivated genes in PCa (626 in total) analyzed using JASPAR2020 database*

**TFs**	**Matrix ID**	TF-BSs in *TET1*-promoter (TSS1500)	TF-BSs in promoters of *TET1*-coactivated genes (TSS1500)		
		N of BSs	Gene N with TF-BSs	Mean N of BSs (SD)	Range of BS N
**ZNF384**	MA1125.1	31	626	43.9 (24.5)	1-161
**NR2C2**	MA1536.1	141	626	29.2 (6.2)	13-49
**ZNF354C**	MA0130.1	42	626	29.1 (8.4)	11-57
**RFX7**	MA1554.1	44	626	28.3 (7.9)	7-66
**MGA**	MA0801.1	17	626	19.1 (5.9)	5-50
**CLOCK**	MA0819.1	15	626	13.9 (5.4)	3-48
**SP3**	MA0746.2	8	625	19 (10.4)	1-68
**ZBTB26**	MA1579.1	9	625	6.7 (2.9)	1-24
**SP1**	MA0079.4	8	624	13.1 (6.3)	1-38
**ZKSCAN1**	MA1585.1	2	624	5.4 (2.5)	1-16
**ZSCAN29**	MA1602.1	7	622	7.3 (3.5)	1-52
**SMAD5**	MA1557.1	5	614	6.8 (3.5)	1-19
**POU2F1**	MA0785.1	10	611	8.3 (11.3)	1-179
**ZBTB6**	MA1581.1	6	611	4.5 (2.4)	1-14
**SP4**	MA0685.1	4	586	5.6 (3.7)	1-27
**ZNF148**	MA1653.1	3	579	9.2 (8)	1-55
**EHF**	MA0598.3	1	561	2.8 (1.5)	1-9
**GABPA**	MA0062.3	1	556	2.6 (1.5)	1-10
**CREB1**	MA0018.4	6	533	2.7 (1.7)	1-10
**RREB1**	MA0073.1	1	383	4 (4.9)	1-55
**REST**	MA0138.2	1	30	1 (0.2)	1-2

Abbreviations and codes: *TET1*: Ten Eleven Translocation family member 1 encoding gene; TFs: transcription factors; PCa: prostate cancer; ID: identification; N: number; BSs: binding sites; SD: standard deviation; TSS1500: 1500 base pairs upstream from transcription start site; *JASPAR2020 database contained data of 21 out of 161 inquired *TET1*-coactivated TFs, and results regarding the 21 TFs are shown; Significances of TF-BSs were calculated according to Touzet and Varre (2007), and statistically significant BSs ($p<0.01$) were considered as true.

Supplementary Table 6 Putative TET1-target genes encoding TFs and showing a promoter hypomethylation and a significantly *TET1*-correlated activation of gene expression in PCa (68 TFs were identified; Promoter methylation in TSS1500 and TSS200 were considered)

TF encoding genes	CpG-probe / ID	Corr. Coeff. CpG-M Gene-E	Corr. p-value CpG-M Gene-E	Corr. Coeff. CpG-M *TET1*-E	Corr. p-value CpG-M *TET1*-E	Diff. methyl. *TET1*-high*	Adj. p-value (Diff. methyl. *TET1*-high)
ADNP	cg05279707	-0.274	2.81E-07	-0.150	5.36E-03	-3.945	6.78E-03
ADNP2	cg14730616	0.017	7.58E-01	-0.186	5.72E-04	-2.873	5.02E-02
ARID2	cg00035453	-0.349	3.14E-11	-0.173	1.38E-03	-2.288	1.21E-01
	cg01353809	-0.229	1.94E-05	-0.123	2.30E-02	-1.385	3.66E-01
ARNT	cg12912293	-0.088	1.05E-01	-0.137	1.14E-02	-2.501	8.95E-02
ASH1L	cg10558228	-0.209	1.03E-04	-0.112	3.92E-02	-0.221	9.07E-01
	cg17038116	-0.058	2.89E-01	-0.119	2.78E-02	-2.284	1.22E-01
BAZ2A	cg18206859	-0.184	6.51E-04	-0.120	2.63E-02	-2.672	6.91E-02
CARF	cg06834261	-0.065	2.31E-01	-0.116	3.27E-02	-1.272	4.11E-01
	cg19847477	-0.080	1.41E-01	-0.107	4.82E-02	-1.546	3.07E-01
EEA1	cg23081353	-0.300	1.56E-08	-0.114	3.47E-02	-2.157	1.45E-01
EHF	cg14022090	-0.112	3.93E-02	-0.108	4.61E-02	-1.782	2.33E-01
HMG20A	cg09419297	-0.270	4.00E-07	-0.248	3.47E-06	-4.306	3.07E-03
	cg17022328	-0.095	7.93E-02	-0.114	3.55E-02	-2.624	7.43E-02
LCOR	cg24296478	-0.303	1.11E-08	-0.151	5.33E-03	-3.852	8.21E-03
NCOA2	cg05868799	-0.029	5.92E-01	-0.115	3.35E-02	-2.431	9.90E-02
NR2C2	cg21255605	-0.180	8.38E-04	-0.197	2.49E-04	-3.470	1.75E-02
REST	cg19490297	-0.292	3.84E-08	-0.266	6.23E-07	-5.102	3.90E-04
RFX7	cg04555097	-0.243	5.49E-06	-0.117	3.02E-02	-1.664	2.69E-01
	cg13935167	-0.274	2.89E-07	-0.221	3.80E-05	-2.804	5.61E-02
	cg19615288	-0.381	3.27E-13	-0.258	1.32E-06	-4.528	1.82E-03
SETBP1	cg13849552	-0.311	4.19E-09	-0.235	1.13E-05	-3.580	1.43E-02
SON	cg00789390	-0.288	6.21E-08	-0.189	4.39E-04	-3.407	1.98E-02
SP1	cg00495415	-0.213	7.20E-05	-0.144	7.61E-03	-2.852	5.18E-02
	cg03437025	-0.114	3.50E-02	-0.110	4.25E-02	-1.031	5.16E-01
SP3	cg14114267	-0.041	4.52E-01	-0.117	3.12E-02	-2.294	1.20E-01
SP4	cg04501438	-0.277	1.97E-07	-0.234	1.25E-05	-4.066	5.22E-03
	cg16721202	-0.081	1.35E-01	-0.108	4.53E-02	-1.339	3.84E-01
USF3	cg17812951	-0.238	8.90E-06	-0.120	2.64E-02	-2.131	1.50E-01
	cg24364084	-0.068	2.08E-01	-0.109	4.45E-02	-0.864	5.95E-01
ZBTB10	cg13562276	-0.317	2.06E-09	-0.124	2.15E-02	-2.073	1.62E-01
	cg27397943	-0.325	7.46E-10	-0.149	5.99E-03	-3.637	1.27E-02
ZBTB11	cg02395779	-0.173	1.36E-03	-0.107	4.90E-02	-1.720	2.51E-01
	cg05405742	-0.191	3.95E-04	-0.162	2.72E-03	-2.627	7.39E-02
	cg05890243	-0.212	8.26E-05	-0.174	1.28E-03	-2.999	4.08E-02
	cg22929104	-0.158	3.38E-03	-0.125	2.09E-02	-2.731	6.30E-02
ZBTB41	cg10127162	-0.333	2.87E-10	-0.192	3.74E-04	-1.520	3.16E-01
	cg16055514	-0.139	1.04E-02	-0.152	4.84E-03	-2.560	8.20E-02
	cg25785281	-0.302	1.30E-08	-0.117	3.11E-02	-2.003	1.78E-01
ZBTB6	cg14582970	-0.115	3.42E-02	-0.122	2.48E-02	-1.080	4.94E-01

ZFP30	cg12019773	-0.232	1.57E-05	-0.163	2.55E-03	-3.473	1.75E-02
	cg26851796	-0.155	4.16E-03	-0.135	1.23E-02	-1.671	2.66E-01
ZFP62	cg11635304	-0.115	3.33E-02	-0.132	1.45E-02	-1.099	4.86E-01
ZFP69B	cg15324448	-0.058	2.84E-01	-0.110	4.27E-02	-2.589	7.84E-02
	cg22375720	-0.113	3.63E-02	-0.189	4.41E-04	-2.973	4.26E-02
ZFP91	cg04570322	-0.262	9.28E-07	-0.155	4.22E-03	-1.941	1.92E-01
	cg05013250	-0.239	8.21E-06	-0.151	5.30E-03	-1.669	2.67E-01
	cg16503683	-0.243	5.73E-06	-0.120	2.64E-02	-0.969	5.45E-01
	cg20957095	-0.217	5.43E-05	-0.126	1.97E-02	-1.752	2.42E-01
	cg23559222	-0.236	1.08E-05	-0.179	9.22E-04	-2.231	1.31E-01
ZHX1	cg14335767	-0.174	1.26E-03	-0.111	4.06E-02	-1.821	2.23E-01
	cg21833640	-0.266	6.22E-07	-0.156	3.95E-03	-1.576	2.97E-01
ZKSCAN8	cg10591475	-0.118	2.95E-02	-0.137	1.15E-02	-2.884	4.93E-02
ZNF107	cg18887107	-0.162	2.76E-03	-0.163	2.53E-03	-2.458	9.52E-02
	cg19933320	-0.259	1.24E-06	-0.255	1.92E-06	-4.903	6.76E-04
ZNF124	cg11979846	-0.202	1.77E-04	-0.146	7.11E-03	-2.562	8.17E-02
ZNF148	cg18107105	-0.226	2.57E-05	-0.193	3.26E-04	-2.502	8.94E-02
ZNF254	cg09777776	-0.206	1.28E-04	-0.144	7.76E-03	-2.602	7.69E-02
ZNF275	cg04754076	-0.153	4.74E-03	-0.257	1.53E-06	-3.902	7.41E-03
ZNF280C	cg04700060	-0.266	6.30E-07	-0.300	1.54E-08	-5.665	7.88E-05
ZNF292	cg00424297	-0.499	0.00E+00	-0.201	1.81E-04	-2.716	6.46E-02
ZNF318	cg25793507	-0.217	5.51E-05	-0.174	1.23E-03	-2.694	6.67E-02
ZNF33A	cg06264303	-0.187	5.18E-04	-0.227	2.27E-05	-4.746	1.02E-03
	cg24602309	-0.078	1.51E-01	-0.119	2.85E-02	-2.922	4.63E-02
	cg27244734	-0.116	3.22E-02	-0.109	4.49E-02	-1.865	2.11E-01
ZNF354C	cg07470489	-0.135	1.25E-02	-0.123	2.35E-02	-1.609	2.86E-01
ZNF384	cg11303425	-0.103	5.64E-02	-0.118	2.97E-02	-2.774	5.88E-02
ZNF41	cg23534593	-0.119	2.82E-02	-0.109	4.35E-02	-1.329	3.88E-01
ZNF417	cg10436115	-0.165	2.18E-03	-0.152	5.04E-03	-2.708	6.53E-02
ZNF426	cg07519373	-0.089	1.00E-01	-0.131	1.58E-02	-1.462	3.37E-01
ZNF493	cg09306584	-0.250	2.92E-06	-0.225	2.80E-05	-4.082	5.05E-03
ZNF507	cg02282382	-0.177	1.06E-03	-0.183	6.90E-04	-4.051	5.40E-03
	cg26813693	-0.189	4.55E-04	-0.177	1.04E-03	-2.990	4.14E-02
ZNF519	cg00691206	-0.141	9.03E-03	-0.199	2.23E-04	-3.024	3.91E-02
ZNF549	cg06458239	-0.159	3.24E-03	-0.115	3.45E-02	-1.800	2.28E-01
ZNF550	cg04958654	-0.482	0.00E+00	-0.118	3.00E-02	-2.495	9.03E-02
ZNF551	cg06615754	-0.122	2.42E-02	-0.114	3.58E-02	-3.449	1.83E-02
	cg22658244	-0.116	3.16E-02	-0.130	1.61E-02	-2.204	1.36E-01
	cg24167603	-0.177	1.02E-03	-0.147	6.72E-03	-2.473	9.32E-02
ZNF558	cg05014724	-0.158	3.55E-03	-0.113	3.75E-02	-1.750	2.42E-01
ZNF573	cg21972176	-0.264	7.40E-07	-0.126	2.01E-02	-2.071	1.63E-01
ZNF611	cg03571507	-0.120	2.68E-02	-0.163	2.60E-03	-2.836	5.33E-02
ZNF615	cg19806182	-0.168	1.89E-03	-0.180	8.38E-04	-3.970	6.40E-03
	cg23224828	-0.187	5.30E-04	-0.146	7.08E-03	-1.441	3.44E-01
ZNF616	cg13735965	-0.131	1.54E-02	-0.114	3.56E-02	-0.451	7.99E-01
ZNF621	cg20978600	-0.191	3.86E-04	-0.138	1.10E-02	-2.904	4.77E-02
ZNF627	cg27594834	-0.262	9.64E-07	-0.240	7.12E-06	-3.888	7.63E-03
ZNF644	cg17170872	-0.226	2.45E-05	-0.109	4.52E-02	-0.795	6.29E-01

ZNF649	cg08052120	-0.269	4.48E-07	-0.117	3.08E-02	-2.668	6.95E-02
	cg15542994	-0.026	6.28E-01	-0.146	6.87E-03	-2.700	6.61E-02
ZNF675	cg25119073	-0.209	9.74E-05	-0.224	3.06E-05	-3.468	1.76E-02
ZNF681	cg25958450	-0.272	3.25E-07	-0.131	1.57E-02		
	cg27517351	-0.324	9.51E-10	-0.166	2.04E-03	-2.888	4.89E-02
ZNF772	cg21489303	-0.276	2.27E-07	-0.112	3.86E-02	-2.078	1.61E-01
ZNF800	cg16738846	-0.189	4.55E-04	-0.138	1.06E-02	-2.897	4.82E-02
ZNF841	cg19012961	-0.263	8.06E-07	-0.283	1.08E-07	-4.833	8.13E-04
ZSCAN23	cg00651523	-0.107	4.85E-02	-0.140	9.64E-03	-0.959	5.50E-01
	cg21486944	-0.077	1.58E-01	-0.110	4.21E-02	-0.899	5.78E-01
ZXDB	cg06776123	-0.285	8.46E-08	-0.138	1.07E-02	-1.565	3.01E-01
ZZZ3	cg01485247	-0.295	2.79E-08	-0.233	1.37E-05	-1.798	2.29E-01
	cg24577193	-0.224	3.02E-05	-0.163	2.47E-03	-3.240	2.69E-02

Abbreviations and codes: TF: transcription factor; TSS: transcription start site; Corr. Coeff. CpG-M Gene-E: correlation coefficient between the CpG-methylation and corresponding gene-expression (Spearman's correlation); Corr. Coeff. CpG-M *TET1*-E: correlation coefficient between CpG-methylation of TF-encoding gene and *TET1*-expression (Spearman's correlation); Diff. methyl.: differential methylation (negative values indicate lower CpG-methylation in *TET1*-high versus *TET1*-low PCa); * differential methylation in *TET1*-high PCa (*TET1* expression above 85. percentile, n=51) versus *TET1*-low PCa (*TET1* expression under 40. percentile, n=136) was calculated by Benjamini-Hochberg method and adjusted p-values are given; green background: significant correlations and differential methylations

Supplementary Table 7 Significantly positive *TET1*-correlated* and *TET1*-coactivated genes in *TET1*-HIGH PCa (n=626, GO, HALLMARK and Canonical Pathway enrichment analysis)

Specific Function	General Function	Gene list
**Enrichment in GO terms "Covalent chromatin modification" "Histone modification" and "Peptidyl-lysine modification"**	**Epigenetic modifiers (n=78)**	*ARID4A; ARID4B; ARNT; ASH1L; ASXL1; ATF7IP; ATM; ATRX; BAZ1A; BAZ1B; BAZ2A; BCOR; BRPF3; CLOCK; CREBBP; CUL4B; DNMT3A; EP300; EP400; EPC1; EPC2; GTF3C4; HMG20A; HUWE1; JARID2; JMJD1C; KANSL1; KAT6A; KAT6B; KDM1B; KDM3A; KDM3B; KDM5A; KDM5B; KDM7A; KIF20B; KMT2A; KMT2C; KMT2D; KMT2E; KMT5B; MAPK8; NIPBL; NOTCH2; NSD1; NUP153; NUP155; NUP160; NUP205; PAXIP1; PIAS1; RESF1; REST; RIF1; RLF; RSF1; SENP1; SENP6; SENP7; SETD2; SETDB1; SIRT1; SMARCAD1; SMC1A; SMC3; STAG1; TAF1; TASOR; TET2; TET3; TMPO; TPR; TRRAP; UBR2; UBR5; USP49; WAC; XPO1*
**Enrichment in Hallmark "Mitotic spindle", "G2M checkpoint" and "E2F targets"**	**Mitotic regulators (n=41)**	*ALMS1; ALS2; ARHGAP5; ARID4A; ATRX; CASP8AP2; CCNT1; CCP110; CEP192; CKAP5; CLASP1; EPB41; IPO7; KIF20B; LATS1; MAPK14; MMS22L; MRE11; NF1; NOTCH2; NUP153; NUP205; ORC2; PMS2; PRKDC; PRPF4B; PURA; RAD50; RASA1; RICTOR; SASS6; SMARCC1; SMC1A; SMC3; SOS1; STAG1; TMPO; TRIO; WRN; XPO1; YTHDC1*
**Enrichment in Canonical pathways „Interaction: P53 regulation", „Reactome: Transcriptional regulation by TP53; Regulation of TP53 activity; Regulation of TP53 activity through phosphorylation; Regulation of TP53 activity through methylation", „Biocarta: P53 hypoxia pathway"**	**P53-related factors**	*ATM; BRPF3; CREBBP; EP300; GATAD2B; JMY; KAT6A; MAPK14; MAPK8; MRE11; RAD50; RICTOR; SUPT16H; TAF1; TAF2; TOPBP1; TTC5; WRN; AGO1; AGO4; APAF1; CCNT1; CCNT2; CDK13; CNOT2; CNOT6L; CSNK1G1; FBXO11; GSK3B; HUWE1; PMS2; TNRC6A*

Abbreviations and codes: PCa: prostate cancer; GO: gene ontology; * strong positive correlation, $p>0.05$, Pearson correlation

Supplementary Table 8 Significantly positive *TET1*-correlated* and *TET1*-coactivated genes in *TET1*-HIGH PCa (n=626, Oncogenic signature gene set enrichment analysis)

Description	GeneRatio	p.adjust	geneID	Count
TBK1.DF_DN	33/320	1.86E-09	*ZZZ3/ACAP2/AKAP9/APPL1/ATG2B/BMPR2/BPTF/CLOCK/DCP2/EHF/ESF1/GTF2A1/MED13L/MIS18BP1/MTR/NF1/RFX7/RIF1/RLF/RSF1/SCAF11/SIKE1/SLK/SMAD5/SMC5/SMG1/SMURF2/SPIN1/TFAM/THOC2/USP46/ZC2HC1A/ZKSCAN1*	33
PGF_UP.V1_UP	24/320	1.16E-07	*ZZZ3/AGL/AKAP9/ATP13A3/BAZ2B/BCLAF1/CEP350/CREB1/DCP2/IREB2/NUP155/OPA1/OSBPL8/PHF3/REV3L/RIF1/SCAF11/SMC3/SP3/TASOR/THOC2/UPF2/WASHC4/ZC3H14*	24
JAK2_DN.V1_DN	17/320	5.93E-05	*ABHD18/ANKRD36B/ATM/CARF/CEP295/CEP97/KMT2A/MBTD1/PHC3/RUFY2/TMEM245/ZBTB44/ZBTB6/ZNF221/ZNF44/ZNF550/ZNF551*	17
BCAT_BILD_ET_AL_DN	9/320	2.41E-04	*ASH1L/FRYL/LNPEP/PRKDC/PUM1/RIF1/SMAD5/SMURF2/ZNF236*	9
VEGF_A_UP.V1_DN	18/320	4.59E-04	*ATP13A3/BAZ1A/BCLAF1/CEP170/MDN1/MFAP3/NUP155/ORC2/PRPF40A/RIF1/SCAF11/SETBP1/TFAM/THOC2/TMPO/TRAPPC8/ZNF124/ZNF253*	18
EIF4E_DN	11/320	4.24E-03	*ARID4B/DIP2B/EP400/MRTFB/OGA/PHIP/PLEKHA5/PRRC2C/UPF2/VPS13C/WDFY3*	11
ERBB2_UP.V1_DN	16/320	5.04E-03	*AGL/C2CD5/FKTN/HAUS3/ICE1/LTN1/RALGAPA1/RIF1/SMC3/TAF2/TOPBP1/ZBTB10/ZBTB11/ZNF107/ZNF43/ZNF430*	16
MTOR_UP.N4.V1_DN	15/320	6.25E-03	*ANKRD36B/ATF7IP/BCOR/CARF/CEP135/CEP295/HBP1/KMT5B/MAML1/MARF1/MIS18BP1/MRE11/SENP6/SENP7/ZNF124*	15
GCNP_SHH_UP_EARLY.V1_UP	14/320	9.40E-03	*ABCB10/ZZZ3/CNOT2/LATS1/MAPK14/MBTD1/MLLT10/MRE11/PUM2/RBBP6/RC3H2/STAG2/STYX/XPO1*	14
RB_P130_DN.V1_DN	11/320	3.06E-02	*AKAP9/APPL1/AVL9/DSP/DSTYK/ESF1/KDM5B/MORC3/PPP4R3B/RASA1/UHRF1BP1L*	11

Abbreviations and codes: PCa: prostate cancer; * strong positive correlation, p>0.05, Pearson correlation

Supplementary Table 9 Significantly positive *TET1*-correlated* and *TET1*-coactivated genes in *TET1*-HIGH PCa (n=626, Univariate Kaplan-Meier survival analysis)

Genes**	p-value	Deaths	Deaths w. Top	Deaths w. Down
CCNT2	0.003	8	7	1
ZNF197	0.009	7	6	1
ORC2	0.011	8	7	1
TOPBP1	0.012	7	6	1
U2SURP	0.012	8	7	1
PWWP2A	0.013	8	7	1
ZNF550	0.015	7	6	1
SMC3	0.016	5	4	1
ZNF782	0.017	8	7	1
MIS18BP1	0.021	9	7	2
ZNF776	0.023	7	6	1
ZNF551	0.024	7	6	1
DNMT3A	0.024	6	6	0
FAM76B	0.025	8	7	1
SLF2	0.027	5	4	1
FANCM	0.027	7	6	1
KIF20B	0.027	6	5	1
HAUS3	0.031	8	7	1
ZNF430	0.033	4	3	1
PIAS1	0.036	5	0	5
MRE11	0.036	7	6	1
ANKRD36B	0.036	6	5	1
ADNP	0.038	6	5	1
ATAD2B	0.038	6	5	1
RAB33B	0.039	5	4	1
HERC4	0.040	6	5	1
MED13	0.041	6	5	1
ZNF558	0.042	8	6	2
RUFY2	0.043	6	5	1
LIMD1	0.046	8	6	2
ANKRD26	0.046	8	6	2
ZNF445	0.047	7	5	2
INVS	0.047	5	4	1
ZNF484	0.048	5	4	1
ZNF841	0.049	8	6	2

Abbreviations and codes: PCa: prostate cancer; * strong positive correlation, p>0.05, Pearson correlation; ** Genes (n=35), whose expression shows a significant association with survival, p-values<0.05 are shown.

Supplementary Table 10 Significantly positive *TET1*-correlated* and *TET1*-coactivated genes in *TET1*-HIGH PCa (n=626, KEGG pathway analysis)

**KEGG Pathway Entry**	**KEGG Pathway Name**	***TET1*-correlated* and -coactivated genes within the indicated pathways**
**hsa05168**	**Herpes simplex virus 1 infection (n=67)**	*APAF1, EIF2AK2, PIK3CA, POU2F1, RBAK, TRAF6, ZFP30, ZFP69B, ZNF100, ZNF107, ZNF12, ZNF124, ZNF141, ZNF17, ZNF221, ZNF253, ZNF254, ZNF275, ZNF320, ZNF33A, ZNF33B, ZNF347, ZNF354C, ZNF41, ZNF417, ZNF426, ZNF43, ZNF430, ZNF44, ZNF484, ZNF490, ZNF510, ZNF519, ZNF543, ZNF548, ZNF549, ZNF550, ZNF551, ZNF558, ZNF573, ZNF585A, ZNF605, ZNF611, ZNF615, ZNF616, ZNF619, ZNF620, ZNF621, ZNF623, ZNF627, ZNF649, ZNF675, ZNF677, ZNF699, ZNF701, ZNF708, ZNF713, ZNF772, ZNF780B, ZNF782, ZNF791, ZNF805, ZNF808, ZNF81, ZNF841, ZNF891, ZNF91*
**hsa00310**	**Lysine degradation (n=9)**	*ASH1L, KMT2A, KMT2C, KMT2D, KMT2E, KMT5B, NSD1, SETD2, SETDB1*
**hsa04550**	**Signaling pathways regulating pluripotency of stem cells (n=13)**	*ACVR2A, ACVR2B, BMPR2, GSK3B, JARID2, KAT6A, MAPK14, PIK3CA, REST, RIF1, SETDB1, SMAD5, SMARCAD1*
**hsa04722**	**Neurotrophin signaling pathway (n=11)**	*BRAF, FRS2, GAB1, GSK3B, KIDINS220, MAP3K1, MAPK14, MAPK8, PIK3CA, SOS1, TRAF6*
**hsa04120**	**Ubiquitin mediated proteolysis (n=12)**	*ANAPC1, BIRC6, CBLB, CUL4B, HERC4, HUWE1, MAP3K1, PIAS1, SMURF2, TRAF6, UBE4A, UBR5*
**hsa04110**	**Cell cycle (n=11)**	*ANAPC1, ATM, CREBBP, EP300, GSK3B, ORC2, PRKDC, SMC1A, SMC3, STAG1, STAG2*

Abbreviations and codes: PCa: prostate cancer; KEGG: Kyoto Encyclopedia of Genes and Genomes, * strong positive correlation, p>0.05, Pearson correlation

Supplementary Table 11 Analysis of PCa and BPH tissue samples, and blood samples from PCa patients with regard to HCMV, EBV, HSV and JCV/BKV virus infection

Material	HCMV	EBV	HSV-1/-2	JCV/BKV
BPH 1	No infection	No infection	No infection	No infection
BPH 2	No infection	No infection	No infection	No infection
BPH 3	No infection	No infection	No infection	No infection
BPH 4	No infection	No infection	No infection	No infection
BPH 5	No infection	No infection	No infection	No infection
BPH 6	No infection	No infection	No infection	No infection
BPH 7	No infection	No infection	No infection	No infection
BPH 8	No infection	779 Genome /mL	No infection	No infection
BPH 9	No infection	No infection	No infection	No infection
BPH 10	No infection	No infection	No infection	No infection
BPH 11	415 Genome/mL	No infection	NA	NA
BPH 12	1430 Genome/mL	No infection	NA	NA
BPH 13	No infection	No infection	NA	NA
BPH 14	No infection	No infection	NA	NA
BPH 15	954 Genome/mL	No infection	NA	NA
BPH 16	No infection	No infection	NA	NA
PCa 1	No infection	No infection	NA	NA
PCa 2	1880 Genome/mL	No infection	NA	NA
PCa 3	No infection	positiv	NA	NA
PCa 4	No infection	No infection	NA	NA
PCa 5	No infection	No infection	NA	NA
PCa 6	No infection	No infection	NA	NA
PCa 7	No infection	No infection	NA	NA
PCa 8	No infection	No infection	NA	NA
PCa 9	23300 Genome/mL	No infection	NA	NA
PCa 10	No infection	No infection	NA	NA
PCa 11	No infection	No infection	NA	NA
PCa 12	No infection	No infection	NA	NA
PCa 13	No infection	No infection	NA	NA
PCa 14	5050 Genome/mL	No infection	NA	NA
PCa 15	No infection	No infection	NA	NA
PCa 16	No infection	No infection	NA	NA
PCa 17	No infection	No infection	NA	NA
PCa 18	14400 Genome/ml	positiv	NA	NA
PCa 19	No infection	No infection	NA	NA
PCa 20	No infection	No infection	NA	NA
PCa 21	62500 Genome/mL	No infection	NA	NA
PCa 22	No infection	No infection	NA	NA
PCa 23	positiv	No infection	NA	NA
PCa 24	No infection	positiv	NA	NA
PCa 25	No infection	No infection	NA	NA
PCa 26	961 Genome/mL	No infection	NA	NA
PCa 27	No infection	No infection	NA	NA

PCa 28	No infection	No infection	NA	NA
PCa 29	129 Genome/mL	No infection	NA	NA
PCa 30	No infection	107 Genome/mL	NA	NA
PCa 31	No infection	No infection	NA	NA
PCa 32	No infection	No infection	NA	NA
PCa 33	No infection	385 Genome/mL	NA	NA
PCa 34	No infection	No infection	NA	NA

Abbreviations: PCa: prostate cancer; BPH: benign prostate hyperplasia; HCMV: human cytomegalovirus; EBV: Epstein-Barr-Virus; HSV-1/-2: herpes simplex viruses 1 and 2; JCV/BKV: human polyomaviruses JC and BK; NA: not analyzed
